# DM9 Domain Containing Protein Functions As a Pattern Recognition Receptor with Broad Microbial Recognition Spectrum

**DOI:** 10.3389/fimmu.2017.01607

**Published:** 2017-11-29

**Authors:** Shuai Jiang, Lingling Wang, Mengmeng Huang, Zhihao Jia, Tobias Weinert, Eberhard Warkentin, Conghui Liu, Xiaorui Song, Haixia Zhang, Jennifer Witt, Limei Qiu, Guohong Peng, Linsheng Song

**Affiliations:** ^1^Key Laboratory of Experimental Marine Biology, Institute of Oceanology, Chinese Academy of Sciences, Qingdao, China; ^2^Liaoning Key Laboratory of Marine Animal Immunology & Disease Control, Dalian Ocean University, Dalian, China; ^3^Paul Scherrer Institute, Laboratory of Biomolecular Research, Villigen, Switzerland; ^4^Department of Molecular Membrane Biology, Max Planck Institute of Biophysics, Frankfurt am Main, Germany

**Keywords:** innate immunity, pattern recognition receptor, DM9 domain, crystal structure, mannose binding, phagocytosis

## Abstract

DM9 domain was first identified in *Drosophila melanogaster*, and it was subsequently found to integrate with or without other protein domains across a wide range of invertebrates and vertebrates. In the present study, a member of DM9 domain containing protein (DM9CP) family from marine invertebrate *Crassostrea gigas* (designated CgDM9CP-1), which was only composed of two DM9 domains, was taken as a protein model to study the biological functions of DM9 domain and its molecular determinants. CgDM9CP-1 was found to exhibit high binding specificity and avidity toward d-mannose residue. It served as a pattern recognition receptor (PRR) with a broad range of recognition spectrum to various pathogen-associated molecular patterns, including lipopolysaccharide, peptidylglycan, mannan, and β-1, 3-glucan in a d-mannose-dependent manner, as well as bacteria and fungi. In order to reveal the molecular mechanism underlying its pattern recognition activity, the crystal structures of wild-type and loss-of-function mutants were solved, and Asp22 and Lys43 were found to be the critical residues for ligand recognition. Moreover, CgDM9CP-1 protein was found to mainly distribute on the surface of *C. gigas* hemocytes, and it could be translocated into cytoplasm and colocalized with the engulfed microbes during hemocyte phagocytosis. The present result clearly indicated that CgDM9CP-1 was a PRR, and it provided an important clue for the better understanding of DM9CP function.

## Introduction

DM9 is a novel protein domain originally identified in *Drosophila melanogaster* with no defined functions (1). Subsequently, Magalhaes et al. characterized a number of toxins, named natterins, from the teleost fish *Thalassophryne nattereri* (2, 3), which contained N-terminal DM9 domains fused to C-terminal Clostridium epsilon toxin/Bacillus mosquitocidal toxin (ETX/MTX2) domains. The cytotoxic activity was probably associated with the ETX/MTX2 domain rather than the DM9 domain, because natterin-like proteins containing only the former toxin domains led to transmembrane pore formation activity, which killed the target cells (4). Recently, increasing DM9 domain containing proteins (DM9CPs) were discovered in both invertebrates and vertebrates. In *D. melanogaster*, a DM9CP (CG16775) was found to be significantly upregulated after oral infection by entomopathogenic *Pseudomonas entomophila* (5), while another three DM9CPs (CG3884, CG10527, and CG13321) were revealed to be part of functional complexes involved in the engulfment of microbial pathogens, intracellular trafficking and phagosome modulation (6, 7). In addition, the expression of a DM9CP named *Plasmodium* responsive salivary 1 (PRS1) was significantly upregulated in the lateral lobe of the salivary glands of *Anopheles gambia* after the invasion of protozoan pathogen *Plasmodium*, and its expression level increased proportionally to the number of infecting sporozoites (8, 9). In vertebrates, DM9CPs were found not only in venomous fish *T. nattereri* but also in viperid snake *Bothrops jararaca*, which could cause cell necrosis, edema, and even permanent disabilities in humans (10, 11). These studies strongly suggest that DM9CPs are participated in the immune response. However, their detailed biological functions and its underlying structural basis are still not well understood.

Pattern recognition is an evolutionarily conserved immune process vital for multicellular organisms to discriminate self from non-self (12, 13). Unlike adaptive immunity with its huge repertoire of lymphoid cell-surface receptors, the innate immune system employs a limited number of receptors, called pattern recognition receptors (PRRs). These PRRs recognize pathogen-associated molecular patterns (PAMPs) presented exclusively on the surface of microorganisms (14). So far, a number of PRRs have been identified with important biological functions in microbial pathogenesis and immune responses (15). For example, some Toll-like receptors (TLRs) and NOD-like receptors recognize various PAMPs through their leucine-rich repeats and modulate downstream inflammatory responses (16, 17). RIG-like helicases act as cytoplasmic sensors for virally derived dsRNA by using helicase domain and subsequently activate antiviral responses (18). PRRs play important roles in the initiation of innate immune defense as well as activation of adaptive immunity through different mechanism (19). For example, some PRRs are mainly associated with signaling activation. The interaction of TLR4 with PAMP ligands can activate the antimicrobial response in macrophages (20). While some other PRRs predominantly function as phagocytic receptors. For instance, when the triggering receptors expressed on myeloid cells 2 was expressed in Chinese hamster ovary cells, it promoted the binding and phagocytosis of *Escherichia coli* and *Staphylococcus aureus* (21). A growing number of PRRs are currently being discovered in both invertebrates and vertebrates, which help us to better understand the molecular mechanisms of microbial pathogenesis and host immune defense.

Recently, Unno et al. reported the isolation and characterization of a DM9CP (designated CgDM9CP-1 in the present study) from marine invertebrate *Crassostrea gigas* (22). It was found to possess high binding specificity toward mannose and high mannose-type N-glycans with the application potential as a research and clinical tool for probing glycans. As a parallel study, we started the project of identification and characterization of DM9CP in early 2013, and the biological function of CgDM9CP-1 was further investigated in the present study with the aim to reveal its role in innate immunity and the potential molecular determinants. CgDM9CP-1 was found to serve as a PRR with extensive microbial binding and agglutination activities. The crystal structure of wild-type and loss-of-function mutants revealed the molecular mechanism underlying its pattern recognition activity. In addition, the molecular phylogeny combined with WebLogo analyses indicated that DM9CPs were ubiquitously distributed and sequence conserved across biological kingdoms, which provided an important clue for the functional study of DM9CP family during the evolution.

## Materials and Methods

### Cultivation of Animals

Adult *C. gigas*, 10–15 cm in length and 150–200 g in weight, were collected from a farm in Qingdao, Shandong Province, China, and acclimated in aerated seawater at 18°C for two weeks prior to use. BALB/C mice were purchased from Qingdao institute for the control of drug products. All the experiments were conducted according to the regulations of local and central government. All animal-involving experiments of this study were approved by the Ethics Committee of Institute of Oceanology, Chinese Academy of Sciences (23).

### The Extraction of Crude Protein from *C. gigas*

The shelled fresh *C. gigas* were crushed and homogenized with a Dounce tissue grinders (Sigma, USA), and 500 g of the wet mass was suspended and extracted with 1,000 ml TBS (50 mM Tris–HCl, pH 7.4, 150 mM NaCl) at 4°C with continuously agitation over night. The extract was centrifuged at 12,000 *g* for 30 min, and the crude proteins in the supernatant were precipitated with 80% (w/v) ammonium sulfate at 4°C over night. The protein precipitate was collected by centrifugation at 12,000 *g* for 1 h, followed by extensively dialysis against TBS for three times at 4°C. The supernatant was collected after centrifugation at 15,000 *g* for 1 h and filtered through a 0.45 µm membrane.

### Preparation of Carbohydrates Coupled Sepharose 6B Matrix

Carbohydrate coupled Sepharose 6B matrix was prepared according to our previous study (24). In brief, epoxy-activated Sepharose 6B matrix (GE Healthcare, Sweden) was washed extensively with distilled water, and then mixed with four kinds of carbohydrates, including d-lactose, *N*-acetyl-d-glucosamine (d-GlcNAc), d-mannose, and l-fucose (Sigma-Aldrich, Buchs, Switzerland), at a final concentration of 200 µM in distilled water (pH 13.0). The mixtures were incubated at 30°C with gentle shaking for 16 h, and 1 M ethanolamine (pH 8.0) was used to block the remaining active groups after TBS washing. The carbohydrate coupled Sepharose 6B matrix was then washed extensively with TBS, and packed into Teflon columns with 100 mm long and 10 mm inner diameter, respectively.

### Purification of Carbohydrate Binding Proteins from *C. gigas*

The d-lactose, d-GlcNAc, d-mannose, and l-fucose coupled Sepharose 6B affinity columns were preequilibrated with TBS, and the crude proteins from *C. gigas* were passed through the carbohydrate coupled Sepharose 6B affinity columns at a flow rate of 1 ml/min on an AKTA avant chromatography system (GE Healthcare, USA), respectively. After extensive TBS washing, the carbohydrate binding proteins were eluted with 200 mM d-lactose, d-GlcNAc, d-mannose, and l-fucose in TBS (pH 7.4) corresponding to each carbohydrate coupled Sepharose 6B column. The eluates were then dialyzed against TBS to remove carbohydrates, and concentrated by a centrifugal filter with 3 kDa cutoff (Millipore, USA).

### Mass Spectrometry Analysis of CgDM9CP-1

For the peptide mass fingerprinting (PMF) analysis, SDS-PAGE was performed to separate the eluate proteins, and the protein bands were visualized by Coomassie Brilliant Blue R-250 (Sigma, USA) staining. The protein band from the d-mannose eluate was excised and tryptically digested, and the peptides were desalted with a ZipTip^TM^ (Millipore, USA) according to the standard protocol (25). MALDI-TOF/TOF-mass spectrometry was performed on an UltrafleXtrem (Bruker Daltonics, Germany). MS spectra were interpreted with the MASCOT software (Matrix Sciences, UK) (26).

### Sequence Retrieval, Domain Prediction and Alignment of DM9CPs

The coding sequence of CgDM9CP-1 was identified by searching the PMF against *C. gigas* genome database. The homology searches of amino acid sequence of CgDM9CP-1 were conducted with BLAST at the National Center for Biotechnology Information (NCBI).^1^ The protein domain was predicted with the simple modular architecture research tool (SMART)^2^ and conserved domain database (CDD) search service.^3^ Multiple alignment was performed with the Clustal X^4^ and the alignment show software Jalview.^5^ Conserved amino acid residues among DM9CPs were identified based on the sequence alignment and presented using WebLogo V3.^6^

### Phylogenetic Analysis

Phylogenetic tree was constructed as previously reported (27). Briefly, the amino acid sequences of DM9CPs were searched against NCBI and the Joint Genome Institute.^7^ Multiple sequence alignments were generated using Clustal W^8^ with default parameters. The alignment was imported into the phylogenetic analysis program MEGA,^9^ and a maximum likelihood tree was generated. A circular phylogenetic tree was then constructed using the interactive tree of life server.^10^

### Cloning of the CgDM9CP-1 Gene

Total RNA was isolated from the hemocytes of *C. gigas* using Trizol reagent (Invitrogen, USA) according to the manufactor’s instruction. RQ1 RNase-free DNase (Promega, USA) was used to digest genomic DNA, and Moloney murine leukemia virus reverse transcriptase (Promega, USA) was used to synthesize cDNA from 1 µg of total RNA. The CgDM9CP-1 coding sequence was amplified using ExTaq polymerase (TaKaRa, Japan) and primers listed in Table 1 with the temperature profile as following: 5 min denaturation step at 95°C and completed by a 10 min extension step at 72°C, with 30 s at 94°C, 20 s at 50°C, and 30 s at 72°C for 30 cycles. The amplified product was purified and cloned into pET-30a vector (Novagen, USA) according to the manufacturer’s instruction. The coding sequence of CgDM9CP-1 was confirmed by sequencing, and the plasmid was then transformed into *E. coli* Transetta (DE3) cells.

**Table 1:** Primers used in the present study.

Primers	Sequence (5′–3′)
**Reverse transcription**	
Oligo (dT)-adaptor	GGCCACGCGTCGACTAGTACT17
Cloning primers for pET-30a (+)	
CgDM9CP-1 F	GGAATTCCATATGGCAGAGTGGGTATC
CgDM9CP-1 R	CCGCTCGAGCTAAATGACTTTATACAG
His-tagged CgDM9CP-1 F	GGAATTCCATATGGCAGAGTGGGTATC
His-tagged CgDM9CP-1 R	CCGCTCGAGCTTAATGACTTTATACAG
**Cloning primers for pET-28a (+)**	
CgDM9CP-1 D22A F	GATACGTGCTGGGTACGCGATTAACAAAAAGGCTTTG
CgDM9CP-1 D22A R	CAAAGCCTTTTTGTTAATCGCGTACCCAGCACGTATC
CgDM9CP-1 K43A F	GAAATGACCCCCGGAGCATGCGGAACCCACC
CgDM9CP-1 K43A R	GGTGGGTTCCGCATGCTCCGGGGGTCATTTC
CgDM9CP-1 H52A F	CACCTCGAAGGGGCAGCAATTCCTTTCGCTGG
CgDM9CP-1 H52A R	CCAGCGAAAGGAATTGCTGCCCCTTCGAGGTG

### Site-Directed Mutagenesis

The mutagenic primers were designed using the PrimerX tool^11^ (Table 1). CgDM9CP-1 mutants, including D22A, K43A and H52A, were constructed by PCR amplification. The coding sequence of mutants were cloned and inserted into the pET28a (+) vector (Novagen, USA), which encoded 6× His-tag at the N-terminal of mutants. All the mutants were confirmed by DNA sequencing, and the plasmid was then transformed into *E. coli* Transetta (DE3) cells.

### Recombinant Protein Expression and Purification

The *E. coli* Transetta (DE3) cells expressing wild-type recombinant CgDM9CP-1 (rCgDM9CP-1) with His-tag were incubated in LB medium at 37°C with shaking at 220 rpm for 3 h. Induction of recombinant proteins was performed with 0.5 mM isopropyl β-d-thiogalactopyranoside (IPTG) at mid-exponential phase (OD 600 nm of 0.4–0.6). The bacteria were then grown at 18°C with shaking at 200 rpm over night. The bacteria was harvested and lysed, and the supernatant was pooled, loaded on to a d-mannose-coupled Sepharose 6B column. rCgDM9CP-1 was eluted using 200 mM d-mannose after extensive washing with TBS. The His-tagged recombinant mutants (D22A, K43A, and H52A) were expressed under the same culture condition with 0.1 mM IPTG induction. The His-tagged recombinant proteins were purified by Ni-NTA affinity chromatography. All the purified proteins were dialyzed over night against TBS at 4°C to remove free ligands.

### Glycan Microarray Analysis

To determine the carbohydrate-binding specificity of rCgDM9CP-1, glycan array screening was performed by the consortium for functional glycomics (Core H)^12^ according to the standard protocol (28). The glycan array (version 5.1) was printed with 610 different natural and synthetic glycans. In the glycan array screening, His-tagged wild-type rCgDM9CP-1 was incubated with slides, and the bound rCgDM9CP-1 was determined by fluorescence labeled anti-His tag antibodies. The fluorescence intensity was detected using a ScanArray 5000 confocal scanner (PerkinElmer, USA). ImaGene image analysis software (BioDiscovery, USA) was used to analyze the image. The relative binding for each glycan was expressed as mean relative fluorescence unit (RFU) of four from the six replicates, with the highest and lowest RFU removed.

### Isothermal Titration Calorimetry (ITC)

Isothermal titration calorimetry experiments on the interaction of rCgDM9CP-1 and its mutants with carbohydrates were performed at 25°C with a VP-ITC isothermal titration calorimeter (Microcal, USA). The freshly purified wild-type rCgDM9CP-1 was dialyzed overnight in PBS (pH 7.4) at 4°C and the protein concentration in the microcalorimeter cell (1.4478 ml volume) was adjusted to 0.05 mM. Carbohydrate solutions, including d-mannose, l-mannose, d-glucose, d-galactose, d-lactose, l-fucose, and d-GlcNAc at a final concentration of 5 mM in PBS were placed in the syringe. After the first injection with 4 µl, 27 injections of 10 µl were conducted with a stirring rate at 300 rpm. The dilution heats of the carbohydrates were measured by injecting different carbohydrate solutions into buffers alone and were subtracted from the experimental curves prior to data analysis. The determination of interactions between mutants and d-mannose were performed by the procedure described above, except that the d-mannose concentration in the syringe was adjusted to 2.5 mM in PBS. The experimental data was fitted to a theoretical titration curve using Microcal ORIGIN software supplied with the instrument, and the standard molar enthalpy change for the binding, $\Delta_{\text{b}}H_{\text{m}}^{0}$, and the dissociation constant, *K*_d_, were derived. The standard molar free energy change, $\Delta_{\text{b}}G_{\text{m}}^{0}$, and the standard molar entropy change, $\Delta_{\text{b}}S_{\text{m}}^{0}$, for the binding reaction were calculated by using the following thermodynamic equations:

(1) $$\Delta_{\text{b}}G_{\text{m}}^{0}=RT\text{ln}K_{\text{d}},$$

(2) $$\Delta_{\text{b}}S_{\text{m}}^{0}=\left(\Delta_{\text{b}}H_{\text{m}}^{0}-\Delta_{\text{b}}G_{\text{m}}^{0}\right)/T.$$

### Crystallization

The purified proteins were concentrated to 10 mg/ml by a centrifugal filter with 3 kDa cutoff (Millipore, MA, USA) at 4°C. The protein precipitate was removed by centrifugation at 12,000 *g* for 30 min, and the supernatant was further filtered through a 0.45 µm filter (Millipore, MA, USA). The protein sample was loaded onto a Superdex 200 10/300 GL gel-filtration column (GE Healthcare, Sweden) equilibrated with TBS at a flow rate of 0.5 ml/min on an AKTA avant chromatography system. The eluates corresponding to the peak areas were collected and used for crystallizations. Standard crystallization screening was carried out using a CrystalMation robot system (Rigaku, USA). Crystallization screens were purchased from Sigma (Sigma, USA), Jena Bioscience (Jena Bioscience, Germany), and Qiagen (Qiagen, Germany). Three hundred nanoliters of protein solution were mixed with crystallization buffer in a ratio of 1:1, sealed and kept at 18°C. Crystallization was done by vapor diffusion in sitting drops. A 300 nl aliquot of wild-type protein rCgDM9CP-1 (dataset name of DM9CPm, 20 mg/ml) was mixed with 300 nl of a reservoir solution composed of 20% (v/v) Poly (ethylene glycol) 2000 monomethyl ether and 100 mM Tris–HCl buffer (pH 7.0). The crystals appeared within 2 days. Crystals of DM9CPm with d-mannose cofactor were obtained within 3 days when adding a 300 nl aliquot of DM9CPm (20 mg/ml) in TBS with 10 mM d-mannose to 300 nl of a reservoir solution composed of 20% (v/v) poly (ethylene glycol) 6000 and 100 mM MES buffer (pH 6.0). For crystallization of D22A mutant (dataset name of DM9CPd), a 300 nl aliquot of DM9CPd (5 mg/ml) in TBS was mixed with 300 nl of a reservoir solution composed of 30% (v/v) poly (ethylene glycol) 3350 and 100 mM HEPES buffer (pH 7.0), and 0.12 M magnisium chloride hexahydrate. The crystal grew within 2 weeks. A 300 nl aliquot of K43A mutant (dataset name of DM9CPk, 5 mg/ml) was mixed with 300 nl of a reservoir solution composed of 25% poly(ethylene glycol) 3350 and 0.1 M Bis-Tris (pH 6.0), in presence of 0.2 M lithium sulfate monohydrate; crystals were obtained after 10 days.

### Data Collection and Processing

Native data were collected at beamline X10SA at the Swiss Light Source (SLS). Long wavelength data for native single-wavelength anomalous diffraction (SAD) phasing were collected at beamline X06DA at SLS. For collection, the crystal was reoriented twice during data collection using the PRIGO mutiaxis goniometer (29). According to the single crystal native SAD phasing strategy described before (30), 3 × 360° long wavelength data were collected at 6 keV, employing phi-fine slicing (31) at three different crystal orientations (chi angles of 0, 10, and 20). Both data sets of mutants D22A and K43A, and native binding with cofactor d-mannose were collected at Beamline X10SA, indexed and processed with the program XDS (32) to a resolution of 1.3, 1.6, and 1.1 Å, respectively.

### Structure Solution

The structure was solved using the SHELXC/D/E pipeline with hkl2map as graphical user interface. Four sites were readily identified with SHELXD with a resolution cutoff of 2.6 Å resulting in CCall and CCweak of 46.0 and 28.5, respectively. Density modification in SHELXE was carried out for 60 cycles using the high resolution native data up to 1.1 Å resolution with a solvent content of 0.45. The initial model was auto built using BUCCANEER and completed with 144 of 144 residues built. The other structures were determined employing the Molecular Replacement Method using the native model DM9CP (PDB ID: 5MH0) as an ensemble in the program PHASER (33).

### Refinement

The structures were refined initially using REFMAC5 (34) and PHENIX REFINE (35) for the final stages. Necessary model improvements as well as search for solvent molecules were performed using COOT (36) and “update water” in PHENIX REFINE. Anisotropic thermal displacement factors were refined at 1.3 Å or better resolution, otherwise using the TLS model. Glycerol was tentatively built in the model to allow for the corresponding electron density as well as d-mannose in the density of DM9CPm.

### Culture of Bacterial and Fungal Cells

*Vibrio splendidus* was grown in 2216E media at 28°C, 220 rpm for 12 h. *E. coli, S. aureus*, and *Bacillus subtilis* were grown in LB media at 37°C, 220 rpm for 8 h. *Pichia pastoris* and *Yarrowia lipolytica* were grown in YPD media at 30°C, 220 rpm for 24 h. All microbes were grown to mid-log phase, harvested by centrifugation at 6,000 *g* for 15 min, and washed three times with PBS.

### Preparation of FITC-Labeled Microbes

Microbes were collected, fixed with 4% paraformaldehyde (PFA) as previously reported (37), and mixed with 1 mg/ml FITC (Sigma, USA) in 0.1 M NaHCO_3_ (pH 9.0) buffer with continuous gentle stirring at room temperature overnight. The FITC-labeled microbes were washed with PBS for three times to eliminate free FITC molecules.

### Microbial Binding and Agglutination Assay

Microbes including *E. coli, V. splendidus, B. subtilis, S. aureus, P. pastoris*, and *Y. lipolytica* (10^8^ cells/ml) were incubated with 3% BSA in PBS for 1 h to block the non-specific binding sites. After three times PBS washing, microbes were incubated with or without rCgDM9CP-1 (control group) at a final concentration of 0.5 mg/ml for 30 min at room temperature. The cells were then washed three times with PBS and incubated with FITC labeled anti-His tag antibody for 1 h at room temperature. After extensive washing, the samples were examined by flow cytometry to detect the microbial binding activity, and analyzed by the fluorescence microscopy to determine the microbial agglutination activity.

### Antibiotic Assay

The antibiotic assay was performed as described previously (38). Briefly, microbes (10^8^ cells/ml) were incubated with 3% BSA in PBS for 1 h, followed by incubation with rCgDM9CP-1 at a final concentration of 0.5 mg/ml at room temperature for 2 h. The cells were then washed three times with PBS and incubated with propidium iodide (5 µg/ml) at room temperature for 10 min. After extensive washing, the samples were examined by flow cytometry.

### PAMP Binding Assay

Pathogen-associated molecular pattern binding activity was determined by modified enzyme-linked immunosorbent assay (ELISA). Briefly, lipopolysaccharide (LPS, from *E. coli* O55:B5, Sigma-Aldrich), peptidylglycan (PGN, from *B. subtilis*, Sigma-Aldrich), mannan (from *Saccharomyces cerevisiae*, Sigma-Aldrich), and β-1,3-glucan (from *Euglena gracilis*, Sigma-Aldrich) were coated on wells of a 96-well microtiter plate (20 μg/well) at 37°C for 1 h. After PBST (PBS, pH7.4, 0.1% Tween-20) washing, the wells were blocked with 3% BSA in PBS at room temperature for 1 h. His-tagged rCgDM9CP-1 was added to each well by incubation at 4°C overnight followed by three times PBST washing. For the carbohydrate inhibition assay, His-tagged rCgDM9CP-1 was preincubated with 200 mM d-glucose, d-GlcNAc, l-fucose, d-lactose, and d-mannose (Sigma-Aldrich, Buchs, Switzerland) for 30 min, respectively, then added to each well of PAMPs coated 96-well microtiter plate and incubation at 4°C overnight followed by three times PBST washing. Horseradish peroxidase (HRP)-labeled anti-His tag monoclonal antibody was added to each well and incubated at room temperature for 1 h. Tetramethylbenzidine substrates (Pierce, Rockford, IL, USA) was added to each well and incubated for 20 min after five times PBST washing, followed by addition of 1 M H_2_SO_4_ to terminate the reaction. The value of each well was recorded at 450 nm by microplate reader (Biotek, USA). The dissociation constant (*K*_d_) was calculated using GraphPad Prism 5 with nonlinear regression curve fit and a one-site binding model analysis. As *A* = *A*_max_ [*L*]/(*K*_d_ + [*L*]), where *A* is the absorbance at 450 nm and [*L*] is the concentration of the rCgDM9CP-1.

### Preparation of Polyclonal Antibodies against rCgDM9CP-1

BALB/C mice were immunized subcutaneously with 50 µg recombinant rCgDM9CP-1 in complete Freund’s adjuvant, and boosted three times in incomplete Freund’s adjuvant at 2-weeks intervals. At 1 week after the final immunization, the blood samples were collected and serum was separated by centrifugation at 2,000 *g*, 4°C for 15 min. The serum titers of the polyclonal antibodies against rCgDM9CP-1 were determined by ELISA. The serum was then buffered by PBS and loaded onto a 2 ml protein A column (GE Healthcare, Sweden). After PBS washing, immunoglobulin G (IgG) was eluted with 100 mM glycine-HCl (pH 2.8). The eluate was rapidly neutralized with 1 M Tris–HCl (pH 8.5), and dialyzed extensively against PBS over night. The eluted IgG was concentrated and the binding specificity toward rCgDM9CP-1 was determined by western blotting.

### Western Blotting Analysis of CgDM9CP-1 Expression

Hemolymph samples from 10 adult *C. gigas* were prepared as described previously (39). After centrifugation at 800 *g*, 4°C for 10 min, supernatant was collected, and hemocytes were pelleted. Hemolymph was centrifuged at 12,000 *g*, 4°C for 10 min to remove cell debris. Hemocytes were washed with PBS (pH 7.2) for three times at 800 *g*, 4°C for 10 min, and lysed in RIPA buffer (50 mM Tris–HCl, pH 7.5, 150 mM NaCl, 1% Nonidet P-40, 0.5% deoxycholate and 0.1% SDS) on ice for 15 min. The cell lysate was collected after centrifugation at 12,000 *g*, 4°C for 10 min. Hepatopancreas, gill, mantle, and adductor muscle from 10 *C. gigas* were collected and homogenized in RIPA buffer using Dounce tissue grinders (Sigma, USA). The supernatant was collected by centrifugation at 12,000 *g*, 4°C for 20 min. The protein concentration was determined by BCA Protein Assay Kit (Pierce, USA). The samples with same amount of proteins (30 µg) were separated by SDS-PAGE. The proteins were transferred from the gel to the polyvinylidene difluoride membranes (Millipore, USA), and the membrane was soaked with 5% BSA in TBST. The membrane was then incubated with antibodies against rCgDM9CP-1 and β-tubulin for 1 h, respectively, followed by HRP labeled secondary antibodies incubation for 1 h. The immune-reactive protein bands were visualized by using an enhanced chemiluminescence kit (Pierce, USA).

### Flow Cytometric Analysis of CgDM9CP-1

Hemolymph was extracted from the posterior adductor muscle sinus using a 2 ml syringe equipped with a 23 G sterile needle, and immediately mixed with pre-chilled anticoagulant citrate dextrose solution A (ACD-A, 0.1 mol/l trisodium citrate, 0.11 mol/l dextrose, and 71 mmol/l citric acid monohydrate) at a volume ratio of 7:1. The hemocytes were harvested by centrifugation at 1,000 *g* at 4°C for 10 min, washed twice, and suspended in modified Leibovitz L15 medium (supplemented with 0.54 g/l KCl, 0.60 g/l CaCl_2_, 1.00 g/l MgSO_4_, 3.90 g/l MgCl_2_, and 20.20 g/l NaCl). After incubation with 3% BSA for 1 h, the hemocytes were incubated with polyclonal IgG against rCgDM9CP-1 for 1 h, while the control hemocytes were incubated with isotype IgG under the same condition. After extensive washing, the hemocytes were incubated with FITC labeled goat anti mouse IgG for 1 h. The fluorescence intensity and the mean fluorescence intensity (10,000 cells) of hemocytes were determined on a FACSAria II flow cytometer (BD Biosciences, USA).

### Confocal Microscopy

Hemocytes were prepared as described above, and plated on glass-bottom culture dishes and incubated at 18°C for 3 h. For the CgDM9CP-1 distribution analysis, 4% PFA was added to fix cells at 4°C for 15 min followed by three times PBS washing, and 0.1% Triton X-100 was added for permialization for 10 min. After PBS washing, 3% BSA in PBS was added to block the nonspecific binding sites for 1 h. Polyclonal IgG against rCgDM9CP-1 were incubated with hemocytes for 1 h, and Alexa Fluor 488 labeled goat antimouse IgG was incubated with hemocytes for 1 h after extensive PBS washing. Hemocytes were further incubated with Dil for 30 min to stain cytoplasmic membrane, and incubated with DAPI for 5 min to stain cell nucleus. For the phagocytosis assay, hemocytes were precultured with FITC labeled microbes for 1 h, and then fixed and permealized as described above. Polyclonal IgG against rCgDM9CP-1 were incubated with hemocytes for 1 h. After PBS washing, Alexa Fluor 594 labeled goat anti-mouse IgG was incubated with hemocytes for 1 h, followed by DAPI staining for 5 min. The hemocytes were monitored and the fluorescent images were taken using Carl Zeiss LSM 710 confocal microscope (Carl Zeiss, Germany).

### Statistical Analysis

The two-sample Student’s *t*-test was used for comparisons between groups. Statistical analysis was performed with GraphPad Prism 5 software. Results are shown as means ± SEM, and statistical significance was defined as *P* < 0.05.

## Results

### The High Binding Specificity and Avidity of CgDM9CP-1 toward d-Mannose

**Figure 1:**
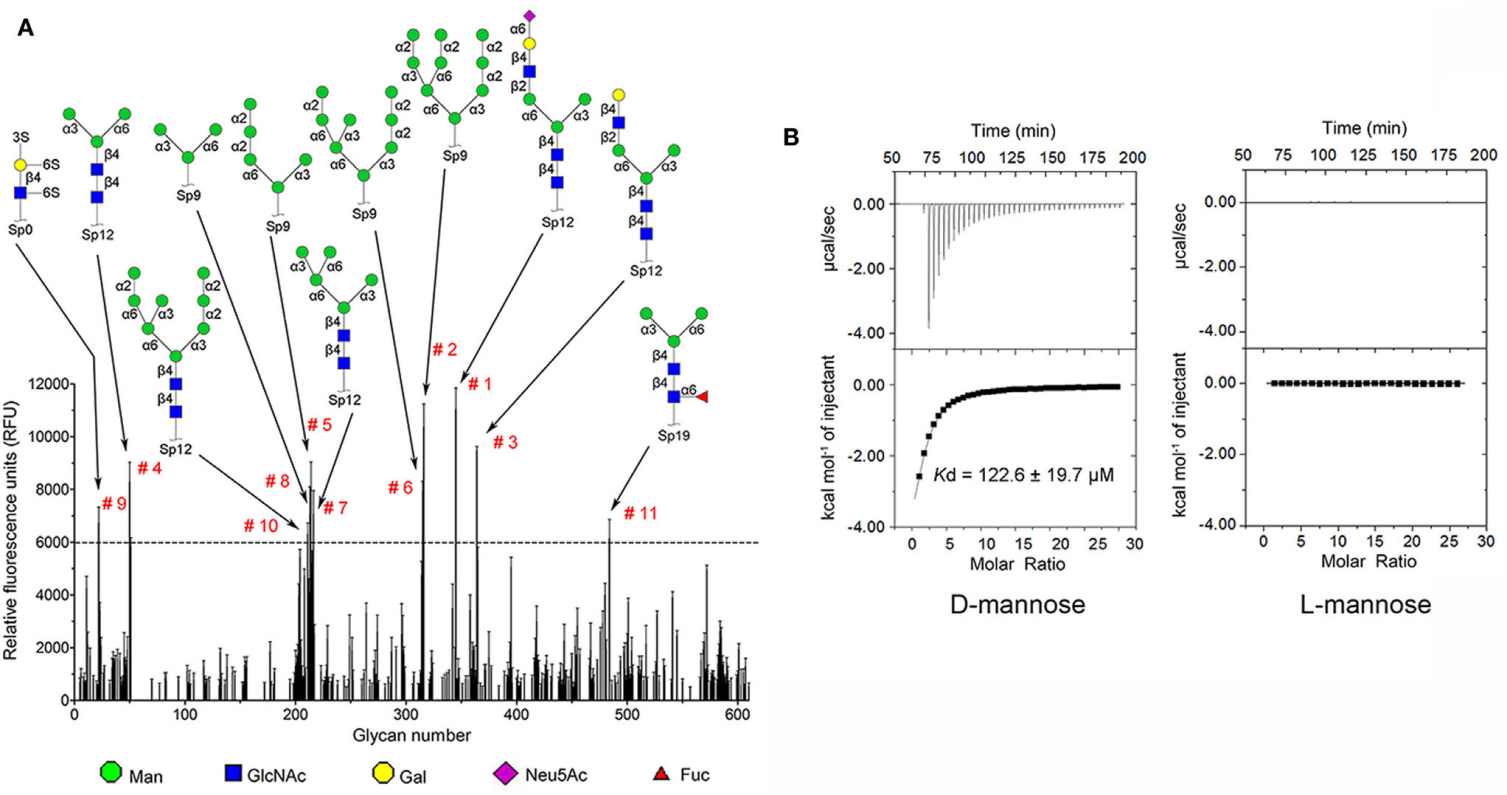
Recombinant *Crassostrea gigas* DM9 domain containing protein 1 (rCgDM9CP-1) exhibited high binding specificity and avidity to d-mannose. **(A)** Glycan microarray analysis (V5.1) was performed to detect the carbohydrate binding specificity. Glycan structures with highest relative fluorescence unit (RFU) are depicted for epitopes. The raw data and the entire list of glycans are available on the consortium for functional glycomics (CFG, http://www.functionalglycomics.org/) website or in Table S2 in Supplementary Material. **(B)** The binding of rCgDM9CP-1 to d-mannose and l-mannose was determined by isothermal titration calorimetry. Upper panel: data obtained from injections of carbohydrate into the protein-containing cell. Lower panel: plot of the total heat released as a function of ligand concentration for the titration shown above (squares). The continuous line represented the best least-squares fit to the obtained data.

Carbohydrate affinity chromatography was employed to isolate glycan binding proteins from crude protein extract of *C. gigas*. A single protein was highly enriched by d-mannose affinity chromatography, whereas d-lactose, *N*-acetyl-d-glucosamine (d-GlcNAc), and l-fucose affinity chromatography yielded far less (Figures S1A,B in Supplementary Material). The d-mannose binding protein was analyzed by MALDI-TOF/TOF-MS, and the amino acid sequence was identified by searching ten tryptic peptides against the genome of *C. gigas* (Figures S1C,D and Table S1 in Supplementary Material). The d-mannose binding protein was identical to the recently reported protein (22). Sequence alignment revealed that CgDM9CP-1 was not homologous to any other known lectins or carbohydrate binding proteins. Protein domain analysis using NCBI’s CDD and SMART showed that CgDM9CP-1 was only composed of two DM9 domains (Figure S2A in Supplementary Material), and they shared 33% sequence identity (Figure S2B in Supplementary Material). The carbohydrate binding specificity of rCgDM9CP-1 was determined by high-throughput glycan microarray printed with 610 different glycans (version 5.1, the consortium for functional glycomics). His-tagged rCgDM9CP-1 was found to bind non-reducing mannosyl glycans with the highest binding specificity. These glycans can be either branched high-mannose oligosaccharides or mannosylated bi- or triantennary hybrid oligosaccharides (Figure 1A; Table S2 in Supplementary Material). The former, for example, glycan #2, #5, #6, and #8, are uniformly composed of mannose residues, while the latter contain at least one branch antenna modified with non-reducing mannose termini, for instance, glycan #1, #3, #4, and #7. rCgDM9CP-1 also exhibited strong binding activity toward glycan #9 [6S(3S)Galβ1-4(6S)GlcNAc], which did not contain mannose residues, suggesting that there might exist another carbohydrate binding mechanism different from that of mannosylated glycans. The carbohydrate binding capacity of rCgDM9CP-1 was further revealed by ITC. The calorimetric data of d-mannose binding was fitted to one binding site model, and the dissociation constant (*K*_d_) was determined to be 122.6 ± 19.7 µM (Figure 1B, left panel and Table 2). Compared with the relatively high binding affinity to d-mannose, rCgDM9CP-1 showed almost no binding affinity to other carbohydrates, including d-glucose, d-galactose, d-lactose, l-fucose, and d-GlcNAc (Figure S3 in Supplementary Material). Moreover, it did not exhibit any binding activity toward the l-isomer of mannose (Figure 1B, right panel).

**Table 2:** Thermodynamic parameters for the binding of rCgDM9CP-1 to d-mannose as determined by ITC.

Thermodynamic parameter (unit)	Value
*N*	1
*K*_a_ (10^3^ M^−1^)	8.16 ± 0.19
*K*_d_ (μM)	122.55 ± 19.68
$\Delta_{\text{b}}H_{\text{m}}^{0}$ (kcal mol^−1^)	−12.01 ± 0.122
$\Delta_{\text{b}}G_{\text{m}}^{0}$ (kcal mol^−1^)	−5.33 ± 0.27
$\Delta_{\text{b}}S_{\text{m}}^{0}$ (cal mol^−1^ K^−1^)	−22.40 ± 1.17

*Thermodynamic parameters, *K*_d_, $\Delta_{b}H_{m}^{0}$ were determined by using the sequential binding sites model. The standard molar binding free energy ($\Delta_{b}G_{m}^{0}$) and the standard molar binding entropy ($\Delta_{b}S_{m}^{0}$) for the binding reaction were calculated using Eqs 1 and 2, respectively. The buffer used in this assay was PBS buffer (pH 7.2), ITC measurements was performed at 25°C. Results are means ± SEM. 1 cal = 4.184 J*.

### Structural Basis for the Specific Binding to d-Mannose

**Figure 2:**
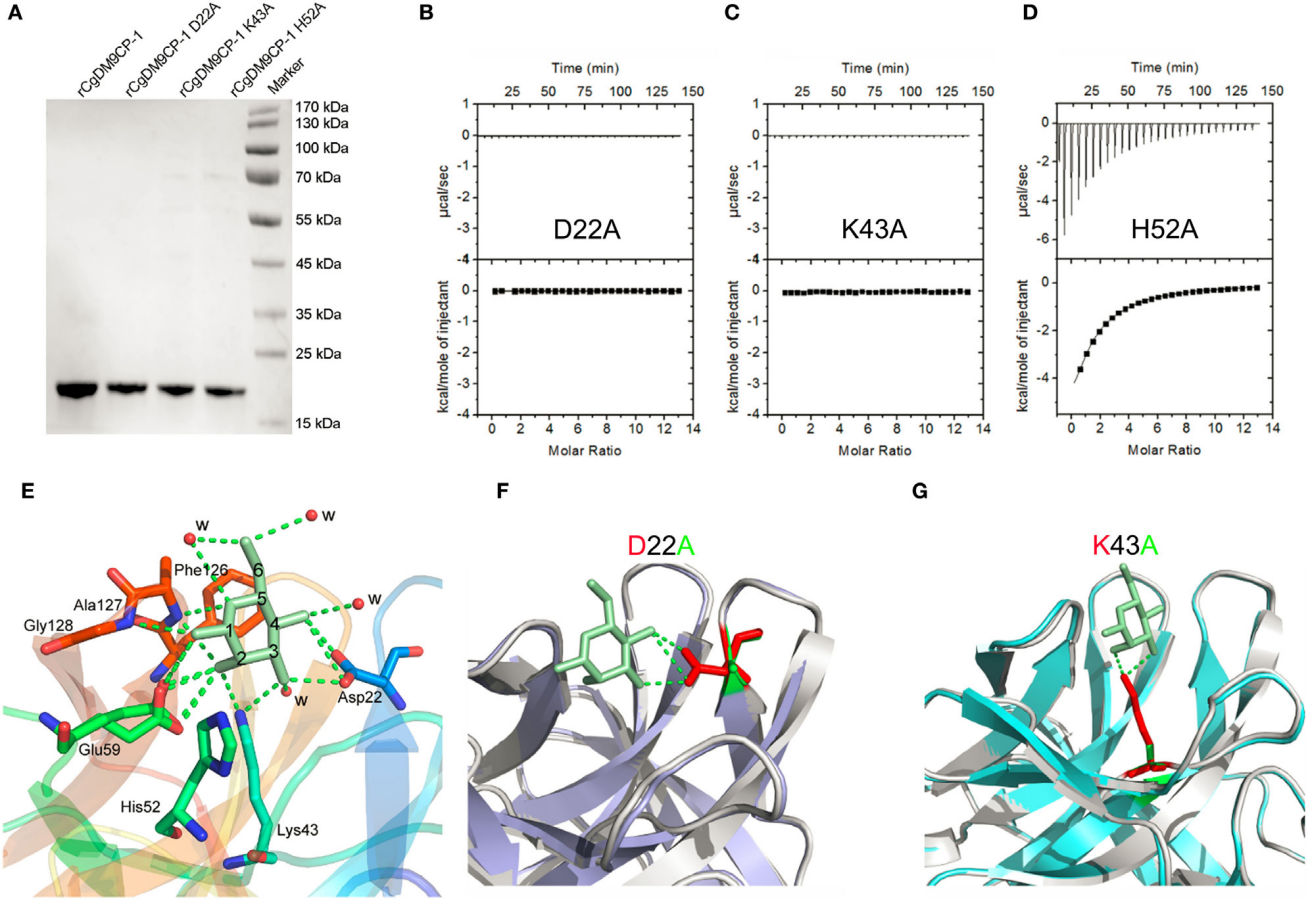
The ligand binding capacity of recombinant *Crassostrea gigas* DM9 domain containing protein 1 (rCgDM9CP-1) and its mutants. **(A)** Purified rCgDM9CP-1 and mutants were analyzed by SDS-PAGE. Isothermal titration calorimetry (ITC) analysis for d-mannose binding to mutant D22A **(B)**, K43A **(C)**, and H52A **(D)** in PBS buffer at 25°C. The crystal structures of wild-type rCgDM9CP-1 in complex with d-mannose [**(E)**, PDB: 5MH1], and the mutants D22A (PDB: 5MH2) and K43A (PDB: 5MH3) are solved. The crystal structures of D22A [**(F)**, purple] and K43A [**(G)**, cyan] were superimposed with that of wild-type rCgDM9CP-1 (gray) in complex with d-mannose (green), respectively. The d-mannose is represented as cyan stick, and the potential amino acid residues involved in the d-mannose binding are shown. The hydrogen bonds between d-mannose and amino acid side chains are represented as dotted lines.

Recombinant CgDM9CP-1 was prepared for crystallization by d-mannose-Sepharose 6B affinity chromatography followed by gel filtration chromatography (Figure 2A; Figure S4A in Supplementary Material). The crystal structure of rCgDM9CP-1 was determined at 1.24 Å resolution using the single crystal native SAD phasing strategy (PDB: 5MH0, Figures S5A,B in Supplementary Material). The diffraction data collection and structure refinement were summarized in Table 3. The crystal structure of rCgDM9CP-1 in complex with d-mannose was solved to reveal the potential residues involved in the d-mannose binding (5MH1, Figure S5C in Supplementary Material), which exhibited similar features to that of native CgDM9CP-1 reported by Unno et al. (22). Moreover, the side chain from Gly128 and four water molecules were also found to participate in the formation of hydrogen bond net work between d-mannose and rCgDM9CP-1 (Figure 2E).

**Table 3:** Data collection and refinement statistics.

Dataset	DM9CP	DM9CP-Sulfur-SAD	DM9CPm	DM9CPd	DM9CPk
**Data collection**					
Space group	P3_2_21	P3_2_21	P3_2_21	P2_1_	P1
Cell dimensions *a, b, c* (Å)	*a* = 67.3, *b* = 67.3, *c* = 55	*a* = 67.3, *b* = 67.3, *c* = 55.0	*a* = 67.3, *b* = 67.3, *c* = 54.8	*a* = 37.16, *b* = 56.53, *c* = 105.53	*a* = 61.93, *b* = 97.96, *c* = 101.42
α, β, *γ* (°)	α = 90.00, β = 90.00, *γ* = 120.00	α = 90.00, β = 90.00, *γ* = 120.00	α = 90.00, β = 90.00, *γ* = 120.00	α = 90.00, β = 93.41, *γ* = 90.00	α = 101.34, β = 107.87, *γ* = 106.46
Temperature (K)	100	100	100	100	100
Wavelength (Å)	0.99999	2.06641	0.99999	0.99999	1.00000
Completeness (%)	82.6 (39.7)	92.0 (46.1)	99.9 (95.9)	98.9 (96.1)	95.5 (89.0)
Redundancy	16.1 (6.3)	23(2.2)	17.0 (14.5)	6.3 (5.8)	3.5 (3.5)
<I/σI>	24.7 (1.2)	56.1 (11.8)	21.7 (1.4)	17.4 (2.5)	16.3 (1.5)
Rmeas (%) (high resolution)	5.7 (199.9)	5.2 (6.7)	7.2 (238)	8.0 (113.6)	6.2 (127.2)
CC(1/2) (high resolution)	100.0 (48.2)	100.0 (99.5)	99.9 (80.6)	99.7 (66.8)	99.9 (52.1)
<d″/sig>		1.5 (0.86)			
No. of S sites		4			

**Refinement statistics**					
Resolution	10–1.24 (1.27–1.24)		2.4–1.1 (1.12–1.10)	20–1.3 (1.33–1.30)	20–1.6 (1.62–1.6)
Rcryst (%)	12.7 (17.7)		10.1 (9.9)	14.5 (26.4)	17.0 (34.2)
Rfree (%)	15.2 (20.9)		11.7 (11.4)	18.2 (30.8)	19.6 (38.5)
R.m.s. deviation bonds (Å)	0.008		0.0088	0.018	0.006
R.m.s. deviation angles (°)	0.92		1.803	1.58	0.8
No. of monomers/AU	1		1	4	12
No. waters/AU	184	1	234	520	2,031
No. glycerol (tentative)/AU	2		1	1	12
No. ligands (d-mannose)/AU			1		

In order to confirm the determinants of interaction between rCgDM9CP-1 and d-mannose, the residues of Asp22, Lys43, and His52 were individually mutated to alanine, and the His-tagged mutants were purified by Ni-NTA affinity chromatography followed by gel filtration chromatography (Figure 2A; Figures S4B–D in Supplementary Material). The mutations D22A and K43A completely abolished the d-mannose binding activities (Figures 2B,C), while the mutation of H52A exhibited a limited effect on the d-mannose binding activity (Figure 2D). The crystal structures of D22A (PDB: 5MH2) and K43A (PDB: 5MH3) were superimposed with that of rCgDM9CP-1 in complex with d-mannose, respectively. Compared with the wild-type protein, both the two mutants exhibited much smaller nonpolar side chains, which significantly impaired the hydrogen bond formation between d-mannose and rCgDM9CP-1 (Figures 2F,G).

### The Extensive Binding Activities of rCgDM9CP-1 toward Microbes and PAMPs

**Figure 3:**
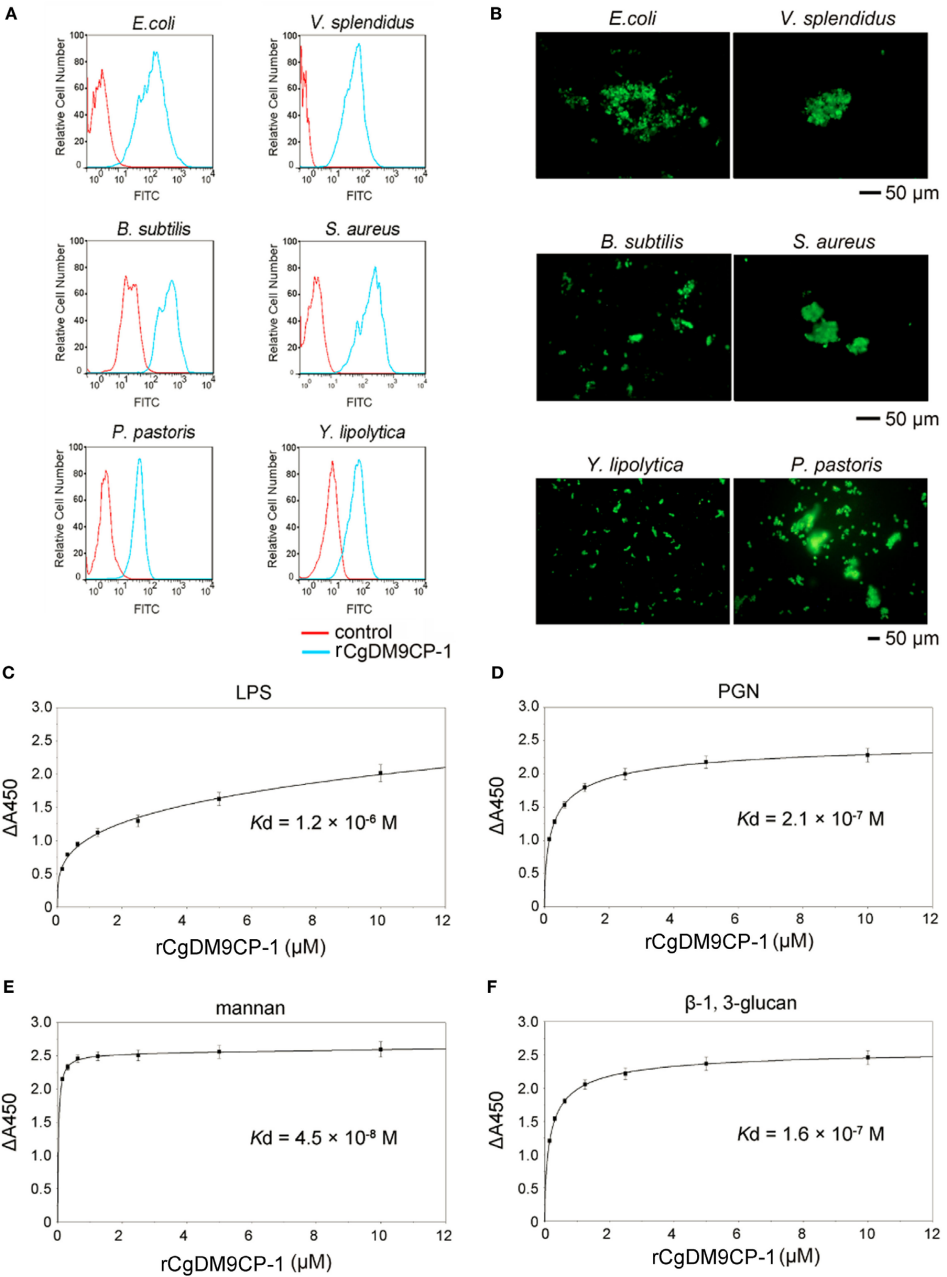
Recombinant *Crassostrea gigas* DM9 domain containing protein 1 (rCgDM9CP-1) exhibited broad microbial and pathogen-associated molecular pattern (PAMP) recognition activity. The microbial binding activity of rCgDM9CP-1 was determined by flow cytometry **(A)**, and the agglutination activity was analyzed by fluorescent microscopy **(B)**. The quantitative binding of rCgDM9CP-1 (0–10 µM) to immobilized lipopolysaccharide (LPS) **(C)**, peptidylglycan (PGN) **(D)**, mannan **(E)**, and β-1, 3-glucan **(F)** was determined by enzyme-linked immunosorbent assay (*n* = 6).

**Figure 4:**
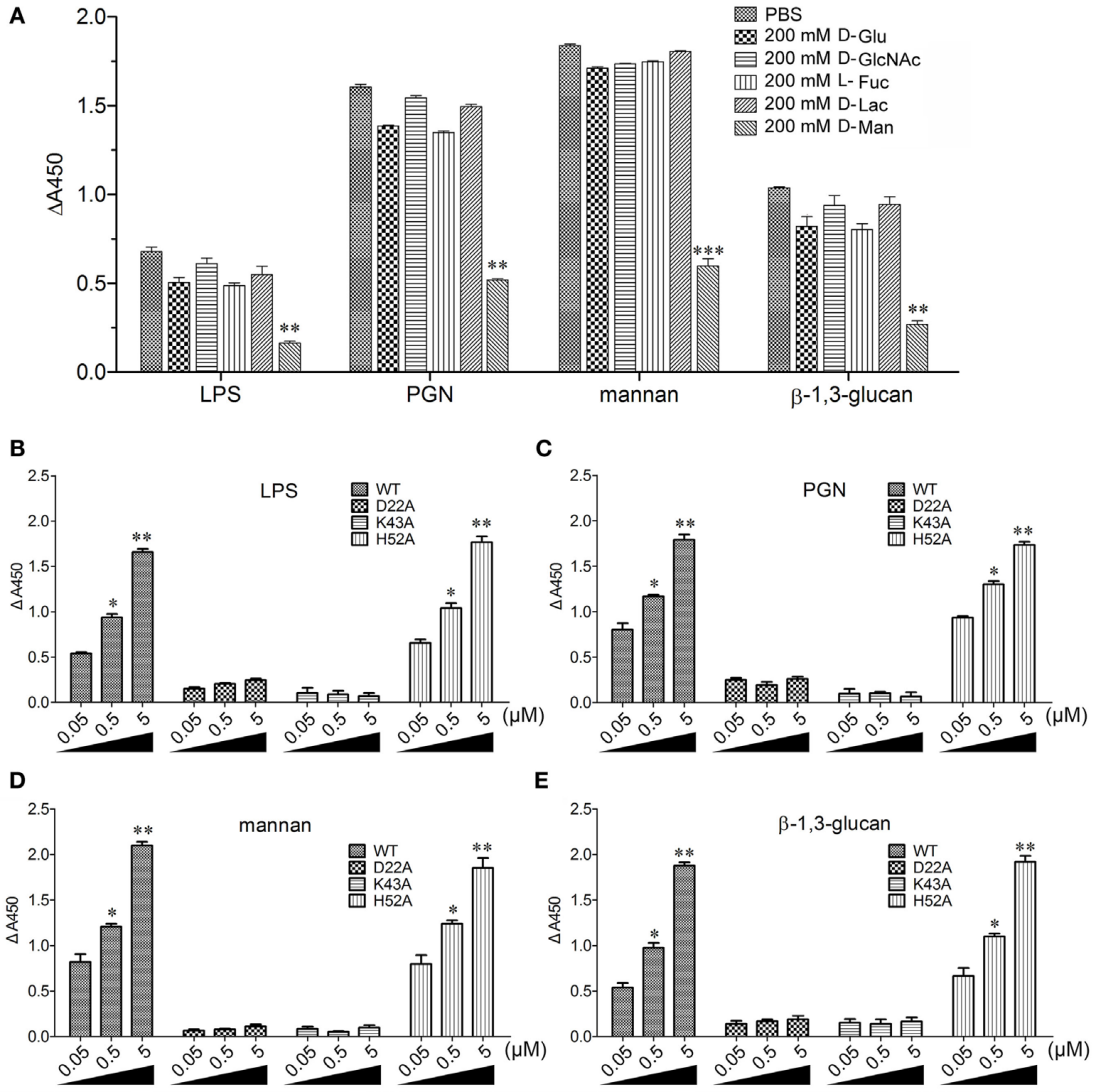
Recombinant *Crassostrea gigas* DM9 domain containing protein 1 (rCgDM9CP-1) binding to pathogen-associated molecular patterns (PAMPs) in d-mannose-dependent manner. **(A)** rCgDM9CP-1 was pre-incubated with 200 mM d-glucose (d-Glu), d-GlcNAc, l-fucose (l-Fuc), d-lactose (d-Lac), and d-mannose (d-Man), respectively, and the binding to PAMPs was determined by enzyme-linked immunosorbent assay (ELISA) (*n* = 6). Data are representative of three independent experiments. *P < 0.05, **P < 0.01, and ***P < 0.001. The quantitative binding of wild-type rCgDM9CP-1 and mutants to immobilized lipopolysaccharide (LPS) **(B)**, peptidylglycan (PGN) **(C)**, mannan **(D)**, and β-1,3-glucan **(E)** was determined by ELISA (*n* = 6). Data are representative of three independent experiments. Error bars indicated standard deviations of the mean. *P < 0.05, **P < 0.01.

The flow cytometric analysis revealed that rCgDM9CP-1 could bind to a number of microbes, including Gram-negative bacteria *V. splendidus* and *E. coli*, gram-positive bacteria *S. aureus* and *B. subtilis*, and fungi *P. pastoris* and *Y. lipolytica* (Figure 3A). Immunofluorescence microscopy further demonstrated the strong microbial binding and agglutination activities of rCgDM9CP-1 toward these microbes (Figure 3B). The binding activities of rCgDM9CP-1 toward different PAMPs, including LPS, PGN, mannan, and β-1,3-glucan, were determined by ELISA. rCgDM9CP-1 could directly bind LPS, PGN, mannan and β-1, 3-glucan in a concentration dependent manner with a saturable process from 0 to 10 µM (Figures 3C–F). The apparent *K*_d_ of rCgDM9CP-1 toward LPS, PGN, mannan and β-1, 3-glucan, calculated from the saturation curve, were 1.2 × 10^−6^, 2.1 × 10^−7^, 4.5 × 10^−8^, and 1.6 × 10^−7^ M, respectively. The binding activities of rCgDM9CP-1 toward LPS, PGN, mannan, and β-1,3-glucan significantly decreased after it was pre-saturated with 200 mM d-mannose (Figure 4A). Correspondingly, the PAMP binding activities of rCgDM9CP-1 significantly changed after the key amino acid residues were mutated. Mutants D22A and K43A showed much lower binding activity toward the four PAMPs even at high concentrations (5 µM), while H52A exerted PAMP binding activity in a concentration dependent manner from 0.05 to 5 µM, which was similar to that of wild-type rCgDM9CP-1 (Figures 4B–E).

### The Involvement of CgDM9CP-1 in Phagocytosis toward Microbes

**Figure 5:**
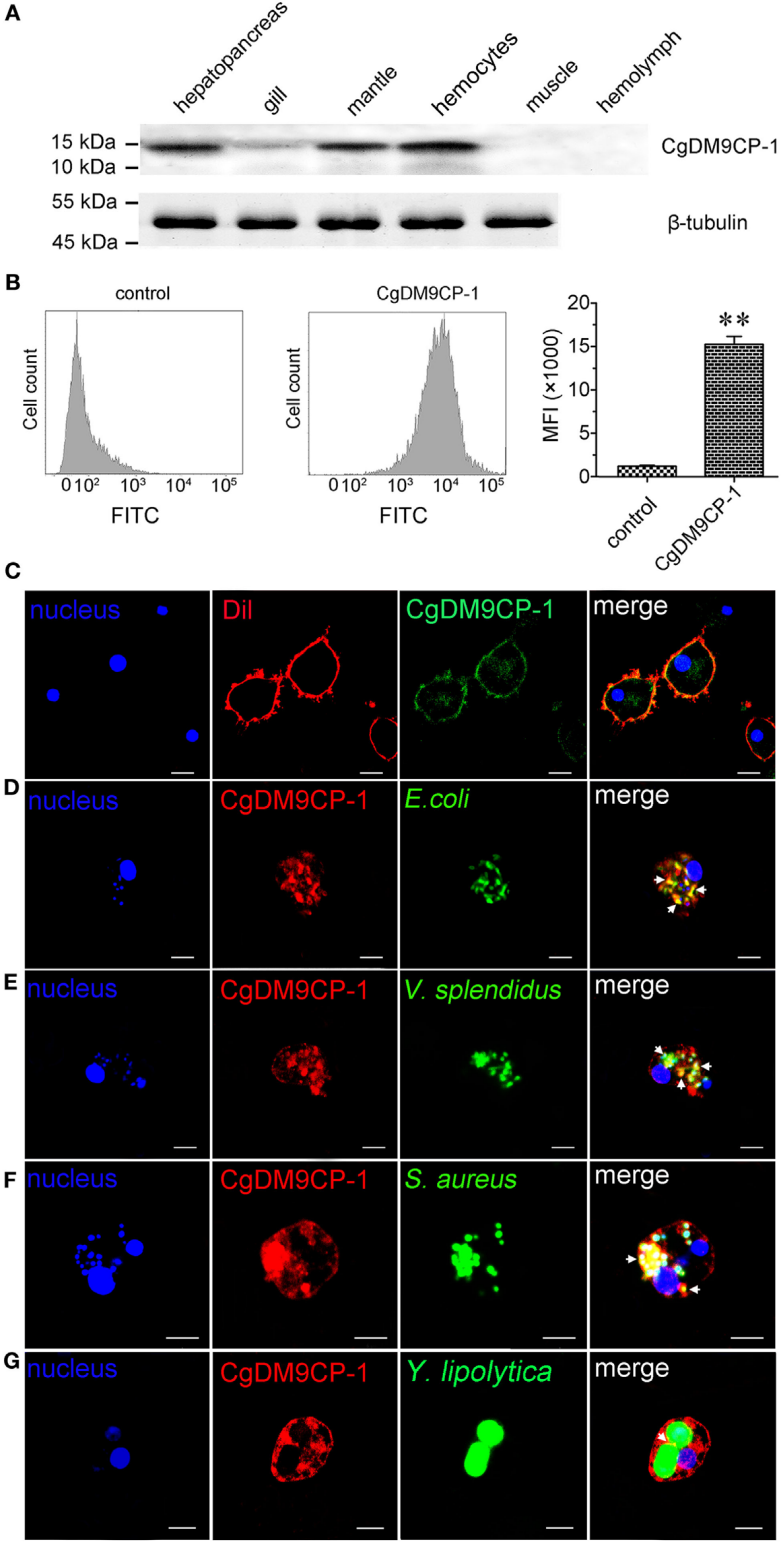
*Crassostrea gigas* DM9 domain containing protein 1 (CgDM9CP-1) was involved in the hemocyte phagocytosis toward microbes. **(A)** The expression profile of CgDM9CP-1 was detected by Western blotting. **(B)** The expression level of CgDM9CP-1 on hemocytes was determined by flow cytometry (*n* = 5), and the data were representative of three independent experiments. **P < 0.01. **(C)** Confocal analysis of CgDM9CP-1 distribution on hemocytes. Hemocytes were stained with polyclonal antibody against CgDM9CP-1 followed by Alexa Fluor 488-labeled antimouse immunoglobulin G (IgG) antibody staining. DAPI and Dil were used to stain cell nucleus and cell membrane, respectively. Hemocytes were incubated with FITC labeled *Escherichia coli* **(D)**, *Vibrio splendidus* **(E)**, *Staphylococcus aureus* **(F)**, and *Yarrowia lipolytica* **(G)** to allow hemocyte phagocytosis, and then stained with polyclonal antibody against CgDM9CP-1 and Alexa Fluor 594 labeled anti-mouse IgG antibody. DAPI was used to indicate cell nucleus. The interactions between CgDM9CP-1 and engulfed microbes were indicated with white arrows **(D–G)**. Bar: 5 µm.

The distribution of CgDM9CP-1 in different tissues was examined by Western blotting. CgDM9CP-1 was highly expressed in hepatopancreas, mantle and hemocytes, lower expressed in gill, while it was hard to be detected in muscle and hemolymph (Figure 5A; Figure S6 in Supplementary Material). Flow cytometric analysis revealed that CgDM9CP-1 was distributed on the outer membrane of hemocytes with high abundance (Figure 5B). Confocal analysis further confirmed that CgDM9CP-1 was mainly distributed on the hemocyte membrane, while less in the cytoplasm under non-challenged condition (Figure 5C). When the hemocytes were incubated with microbes, including *E. coli, V. splendidus, S. aureus*, and *Y. lipolytica*, to induce phagocytosis, CgDM9CP-1 could internalize from the cell membrane into the cytoplasm, and was found to colocalize with or surround the engulfed microbes (Figures 5D–G). Moreover, CgDM9CP-1 was found to exhibited antibiotic activity toward *Y. lipolytica*, while its antibiotic activity toward Gram-negative and Gram-positive bacteria was weak (Figure S7 in Supplementary Material).

### DM9CPs are Ubiquitously Distributed and Sequence Conserved

**Figure 6:**
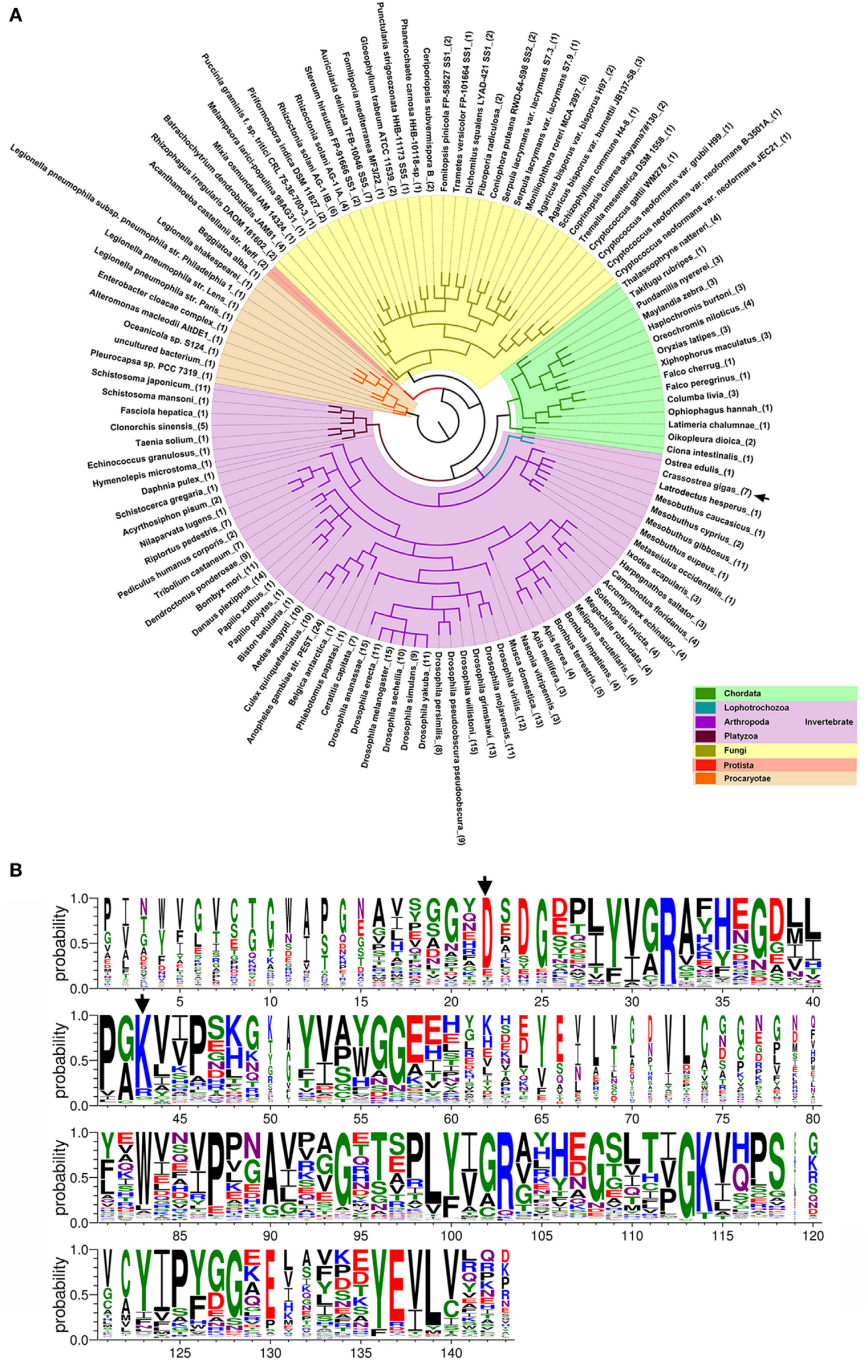
Conserved features of DM9 domain containing proteins (DM9CPs). **(A)** Maximum likelihood phylogenetic tree of 477 DM9CPs annotated from the released genomes. Procaryotae, Protista and Fungi Kingdom are color-coded with brown, red, and yellow, respectively. The invertebrates and chordats in Animalia kingdom are color coded with purple and green, respectively. The DM9CPs in *Crassostrea gigas* were indicated by a black arrow. **(B)** WebLogo sequence alignment of DM9CP proteins identified with BLASTP. CgDM9CP-1 and another 476 annotated DM9CPs were used as input for sequence comparison. The residues (Asp22 and Lys43) involved in the ligand binding were highly conserved and marked by black arrows.

To date, up to 477 DM9CPs have been annotated in the released genomes of a wide range of organisms from the Procaryotae, Fungi, Protista and Animalia Kingdoms (Figure S8A and Table S3 in Supplementary Material). DM9CPs are found to be of multi-copy in animal species, especially in invertebrates, with approximately ten similar genes in each *Drosophila* species (Figure 6A; Figure S8B in Supplementary Material). In *C. gigas*, seven DM9CPs were annotated, and they all contained two DM9 domains and shared high similarity with each other (Figures S2C,D in Supplementary Material). In order to further analyze the potential biological function of DM9CP family members, CgDM9CP-1 was aligned with another 476 DM9CPs using WebLogo (Figure 6B). The primary structure alignment illustrated that the amino acid sequence of DM9 domains were highly conserved throughout evolution, such as Asp22 and Lys43, which were found to be essential for the ligand binding in CgDM9CP-1.

## Discussion

The potential biological functions of DM9 domain have been reported in several invertebrates and vertebrates. In invertebrates, a DM9CP (CG16775) from *D. melanogaster* was found specifically upregulated after oral infection by microbial pathogens but not septic injury with unknown functions (5). A system biology analysis of phagosome combined with the protein interaction network of *D. melanogaster* revealed that three DM9CPs (CG3884, CG10527, and CG13321) could interact with other proteins to form functional complexes involved in phagocytosis of microbial pathogens and phagosome modulation in the innate immunity (6, 7). A DM9CP named PRS1 was predominantly expressed in distal part of the lateral lobe of salivary glands, where the protozoan pathogen *Plasmodium* infected the *A. gambia* (9). After infection, the expression level of PRS1 was upregulated not only in salivary glands but also in midgut epithelial cells, which were the critical barriers for the pathogens to pass through to develop in mosquitoes. In addition, PRS1 was found to be highly concentrated into vesicle-like structures in infected cells (8). Another three DM9CPs with high similarities have been identified in human liver fluke, and two of them from liver fluke *Fasciola hepatica* and *Opisthorchis viverrini*, respectively, were both highly distributed on the surface of the tegument, which was the outermost surface and major interface between host and environment (40, 41). The other DM9CP from *Fasciola gigantic* was found to localized into cytoplasmic vesicle-like structures after the infection of bacteria, which was similar to that of PRS1 (42). In vertebrates, two toxins containing DM9 domains were found to be highly expressed in venomous fish *T. nattereri* and snake *B. jararaca* (10, 11). Increasing evidences suggested that DM9CPs played important roles in the host immune responses. However, the biological function of DM9CPs in innate immunity still remains largely unknown.

Recently, CgDM9CP-1 was isolated and characterized as a member of lectin family (*C. gigas* lectin 1) with the application potential as a research and clinical tool for probing glycans (22). Structurally, CgDM9CP-1 is only composed of two DM9 domains without any other domain, which provides an ideal protein model to study the biological function DM9 domain. The amino acid sequence analysis revealed that CgDM9CP-1 neither showed any homology to the known lectins nor contained any lectin domains or known carbohydrate binding motifs, but exhibited high similarity and identity to other DM9CP family members (Figure 6B). More pronounced, CgDM9CP-1 exhibited strong binding activities and broad binding spectrum toward different PAMPs as well as microbes, which was essential for pattern recognition of microbes during innate immune response. In addition, a number of immune receptors with carbohydrate binding activities are designated according its biological functions. For example, dendritic cell specific intracellular adhesion molecule-3 grabbing nonintegrin (DC-SIGN), which represented a mannose binding C-type lectin, was proved to be an important PRR toward various types of pathogens and involved in the modulation of immune responses of dendritic cells (43, 44). Langerin, myeloid C-type lectin presented on the cell surface of Langerhans cells, was functioned as a PRR possessing broad microbial recognition spectrum with highly diverging avidity and selectivity (45, 46). Fibrinogen-related protein 3 with high binding specificity to galactose was involved in the microbial phagocytosis in the snail *Biomphalaria glabrata* (47). Collectively, our findings strongly support that DM9CPs represent a novel type of PRR family, which sheds new light on the functional study of DM9CPs in the innate immunity.

Mannose binding receptors have been reported to play important roles in the pattern recognition. For example, the macrophage mannose receptor could recognize mannan presented on fungal pathogens, and activate the Th17 signaling pathway during pathogen-specific host immune response (48). Dectin-2, a direct receptor for mannose-capped lipoarabinomannan, could induce pro- and anti-inflammatory cytokines production through Dectin-2-FcRγ signaling axis in the autoimmune encephalitis disease (49). In the present study, the glycan microarray with much higher throughput, which was complementary to that reported in Unno’s study, showed that CgDM9CP-1 possessed high binding specificity toward mannosylated glycans. The association constant (*K*_a_) of rCgDM9CP-1 interaction with d-mannose was determined to be 8.16 ± 0.19 × 10^3^ M^−1^ (Table 2), while it was 2.00 ± 0.46 × 10^3^ M^−1^ reported by Unno et al. (22). The difference of the *K*_a_ might due to the protein post-translational modification of native protein, for example, N-terminal of native CgDM9CP-1 was found to be modified by the acetyl group (22). Similar to the pattern recognition activities of known mannose receptors, rCgDM9CP-1 exhibited strong binding activity toward mannan. Moreover, rCgDM9CP-1 also displayed strong pattern recognition activities toward LPS, PGN, and β-1,3-glucan, indicating a more extensive microbial binding profile than that of previously reported mannose binding receptors. In *C. gigas*, CgDM9CP-1 was highly expressed in the tissues important for pathogen recognition and innate immune defense such as hepatopancreas, mantle and hemocytes (50), while not in hemolymph, suggesting that CgDM9CP-1 was not an opsonin. When the hemocytes encountered with microbes, CgDM9CP-1 was internalized from the cell membrane into cytoplasm accompany with the hemocyte phagocytosis, and colocalized with the engulfed microbes, indicating that CgDM9CP-1 was involved in the direct interaction with microbes and modulation of hemocyte phagocytosis. It has been reported that some cell surface bound PRRs can recognize its ligand and activate the intracellular signaling pathway to provoke potent immune responses against pathogens, while some other cell membrane PRRs mainly function as phagocytic receptor involved in the modulation of phagocytosis of microbial pathogens (51, 52). Herein, CgDM9CP-1 seemed to act mainly as an immune receptor involved in the hemocyte phagocytosis in the innate immunity of *C. gigas*. Although CgDM9CP-1 lacked the transmembrane domain, the cell surface distribution was probably due to the interaction of CgDM9CP-1 with other membrane cofactors. For example, cell membrane protein CD14 without a transmembrane domain was found to locate on the cell membrane *via* the linkage to glycosylphosphatidylinositol (53). MD2 is an important immune receptor that proved to distribute on the cell membrane through the interaction with integral membrane protein TLR4 (54, 55). The potential molecular mechanism of CgDM9CP-1 membrane localization remains to be investigated.

As a previously unidentified PRR, CgDM9CP-1 displayed its PAMP recognition activity with a distinctive structural basis. In the previous study, CgDM9CP-1 was computationally determined to be dimeric state using the PISA program (proteins, interfaces, structures, and assemblies). Herein, the molecular weight of rCgDM9CP-1 was calculated to be approximately 17 kDa according to the main elution peak (about 17.5 ml elution volumes) from gel filtration chromatography, which is similar to the theoretical monomeric protein mass (about 16.5 kDa). Western blotting analysis revealed that there existed both monomeric and dimeric state of rCgDM9CP-1, and the abundance of monomeric form was much higher than that of dimeric form (data not shown). The results collectively indicated that rCgDM9CP-1 existed as both monomeric and dimeric form with different abundances. In order to reveal the molecular determinants underlying its pattern recognition activity, the crystal structures of rCgDM9CP-1, the corresponding mutants, and the complex with d-mannose ligand were solved using the single crystal native SAD phasing strategy. The crystal structure analysis revealed that rCgDM9CP-1 did not show any three dimensional homology to other known PRRs. In our present study, the ligand binding site was located on the boundaries between two DM9 domains, and the amino acid residues from two DM9 domains assembled together to form the ligand recognition motif. The side chains from Asp22 and Lys43 residues were found to be essential for the d-mannose and PAMP binding, and the mutation of these two amino acid residues significantly abolished the PAMP binding activity. Although the side chain of His52 was supposed to be involved in the stacking interactions between the hydrophobic portion of d-mannose to stabilize the carbohydrate binding activity, the mutation H52A did not exhibit significantly decrease of d-mannose or PAMP binding activity, indicating that His52 might not be an essential residues in the pattern recognition.

DM9 domain has been found to exist in various proteins from a number of species. The existence of DM9CPs in prokaryotic cells, such as *Legionella pneumophila* and *Enterobacter cloacae*, indicates that the DM9 domain is an ancient protein domain probably evolved from prokaryotes (56). Although DM9 domains are extensively found in vertebrates, such as bony fishes, reptiles and birds, it is quite unexpected that the domain is absent in mammals. It is also noteworthy that DM9CPs have not been identified in the Plantae Kingdom, suggesting that this protein domain probably has been lost during evolution. The uneven distribution of phylogenetic patterns of the DM9 domain is likely to reflect the natural selection during molecular evolution in innate immunity. Moreover, DM9 domains are usually found to exist as tandem arranged repeats in proteins, especially in different *Drosophila* species (Figure S9 in Supplementary Material), which might contribute to the enhanced ligand binding activities. Interestingly, there are a number of DM9 domains which are fused with other protein domains. For example, the natterins reported in *T. nattereri* fish and viperid snake *B. jararaca* contain both DM9 domain and pore-forming ETX/MTX2 domain (10, 57). In desert locust *Schistocerca gregaria* and the Mediterranean fruit fly *Ceratitis capitata*, DM9CPs were found to contain both DM9 domains and farnesoic acid O-methyl transferase domains (58, 59). The fusion of DM9 domains with other domains suggested that these proteins probably exerted multiple biological functions involved in different biological pathways.

## Ethics Statement

All animal-involving experiments of this study were approved by the Ethics Committee of Institute of Oceanology, Chinese Academy of Sciences.

## Author Contributions

SJ performed the native protein purification and glycan array analysis. SJ, ZJ, and XS performed cloning work, expression, and purification of recombinant proteins. MH, HZ, and LQ performed molecular interaction and microbial binding experiments. SJ, MH, JW, and GP crystallized the proteins. TW, EW, and GP solve the crystal structure. SJ and CL constructed the phylogenetic tree. SJ, LW, GP, and LS design research and wrote the manuscript.

## Conflict of Interest Statement

The authors declare that the research was conducted in the absence of any commercial or financial relationships that could be construed as a potential conflict of interest.

## Footnotes

^1^https://blast.ncbi.nlm.nih.gov/Blast.cgi.

^2^http://smart.embl-heidelberg.de/.

^3^http://www.ncbi.nlm.nih.gov/Structure/cdd/wrpsb.cgi.

^4^http://www.clustal.org/clustal2/.

^5^http://www.jalview.org/.

^6^http://weblogo.berkeley.edu/logo.cgi.

^7^http://genome.jgi-psf.org/Brafl1/Brafl1.home.html.

^8^http://www.clustal.org/.

^9^http://www.megasoftware.net/.

^10^http://itol.embl.de.

^11^www.bioinformatics.org/primerx.

^12^http://www.functionalglycomics.org/.

## Abbreviations

PRR, pattern recognition receptor; PAMP, pathogen-associated molecular pattern; DM9CP, DM9 domain containing protein; ITC, isothermal titration calorimetry.
