# Supplementary material for: DM9 Domain Containing Protein Functions As a Pattern Recognition Receptor with Broad Microbial Recognition Spectrum
Source: Front Immunol. 2017 Nov 29;8:1607. doi: 10.3389/fimmu.2017.01607 (PMC5712788; doi:10.3389/fimmu.2017.01607)
Supplement: Supplementary file 1 [file Image_1.pdf]

SUPPLEMENTARY FIGURES

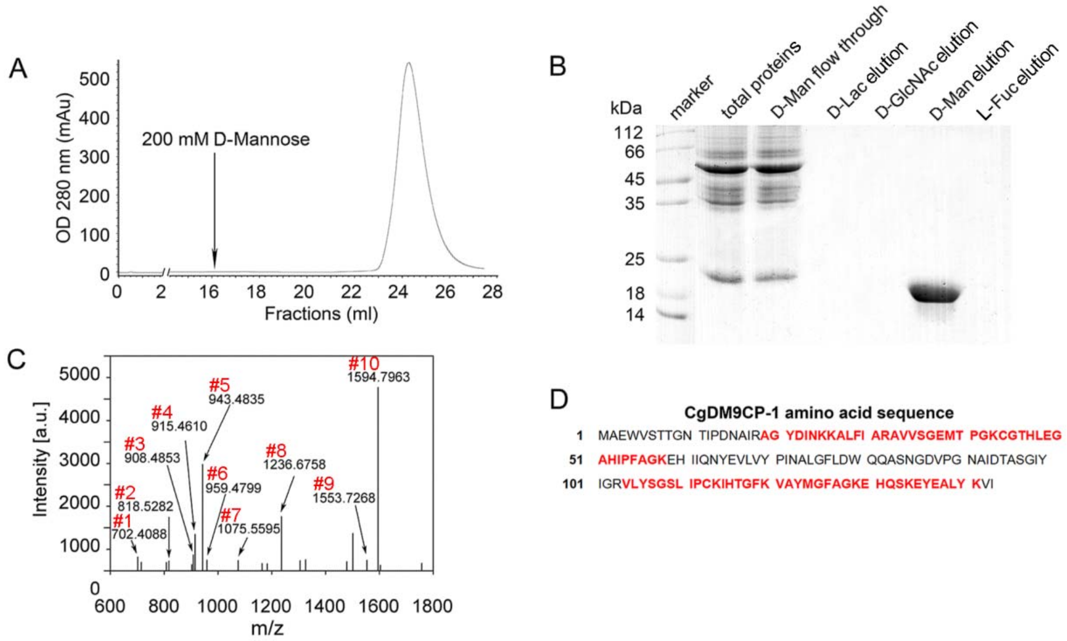

FIGURE S1 Purification and identification of a D-mannose binding protein from *Crassostrea gigas*. Crude protein extract of *C. gigas* were subjected to carbohydrate affinity chromatography. D-mannose-Sepharose 6B highly enriched glycan binding proteins (**A**), and the eluate yield a single protein band by SDS-PAGE analysis (**B**). The total proteins and flow through of D-mannose-Sepharose 6B chromatography were separated by SDS-PAGE. The carbohydrates used in carbohydrate affinity chromatography are D-lactose (D-Lac), *N*-Acetyl-D-glucosamine (D-GlcNAc), D-mannose (D-Man) and L-fucose (L-Fuc). The D-mannose binding protein was identified by MALDI-TOF/TOF-MS, and peptide mass fingerprinting (PMF) is shown. Peptides were labeled with numbers according to the peptide mass (**C**). Ten tryptic amino acid sequences were identified and showed in red bold font with 54.55% coverage of the full length CgDM9CP-1 (**D**).

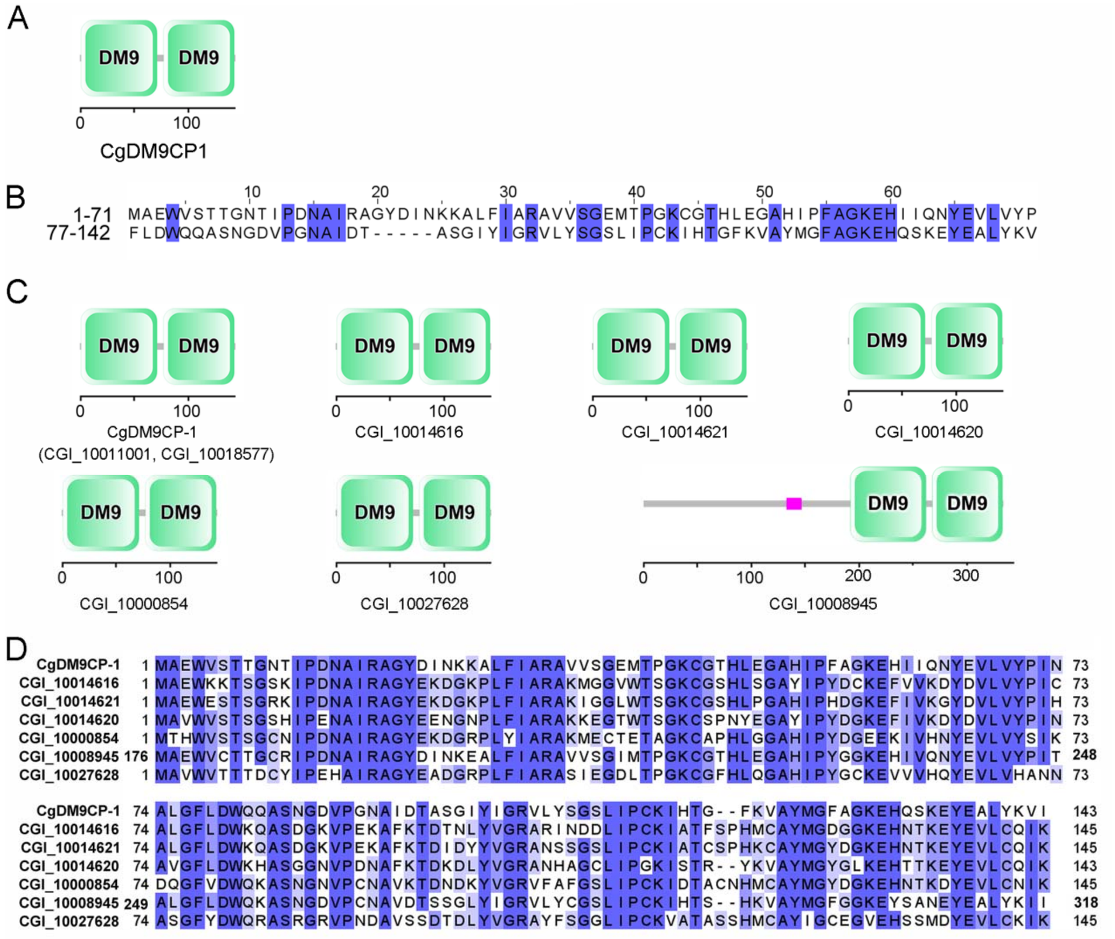

FIGURE S2 Protein domain analysis of CgDM9CP-1. (**A**) CgDM9CP-1 is composed of two DM9 domains. (**B**) Two DM9 domains were located at the positions from 1 to 71 and 77 to 142 amino acid respectively, which share 33% sequence identity. The conserved residues were shaded. (**C**) Totally seven DM9CPs were identified in the *C. gigas* genome database. (**D**) The primary structures of *C. gigas* DM9CPs share high sequence similarity and identity. The conserved residues were shaded.

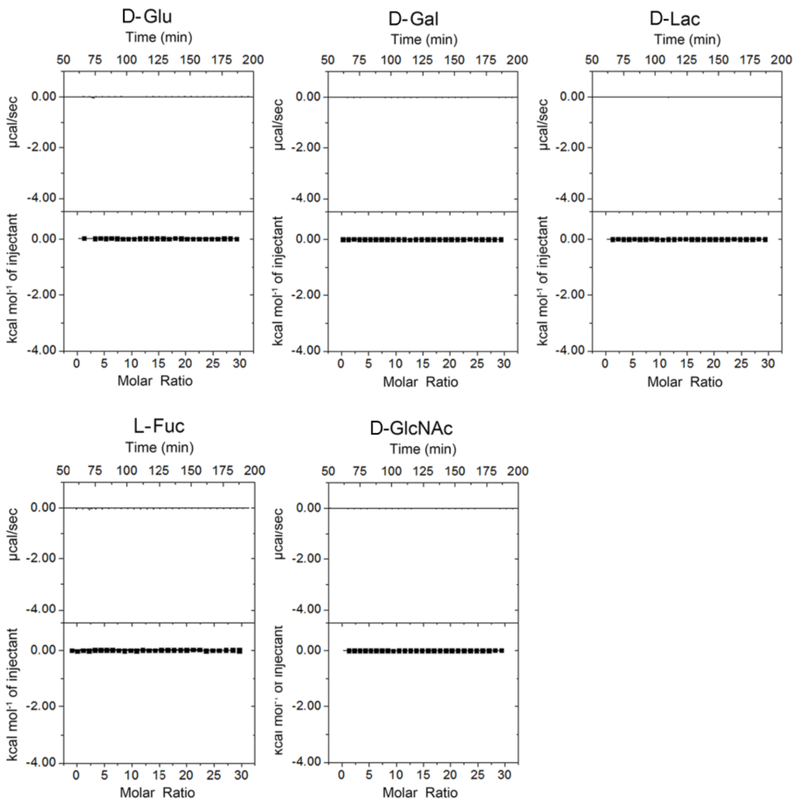

FIGURE S3 The interactions of rCgDM9CP-1 towards different carbohydrates detected by isothermal titration calorimetry (ITC). ITC data obtained by injecting different carbohydrates (5 mM) into a solution of rCgDM9CP-1 (0.05 mM) at 25 °C respectively. Upper panel: data obtained from 27 automatic injections of 10 µl of carbohydrate each into the protein-containing cell. Lower panel: plot of the total heat released as a function of ligand concentration for the titration shown above (squares). The continuous line represented the best least-squares fit to the obtained data. The carbohydrates used in ITC are D-glucose (D-Glu), D-galactose (D-Gal), D-lactose (D-Lac), L-fucose (L-Fuc) and *N*-Acetyl-D-glucosamine (D-GlcNAc).

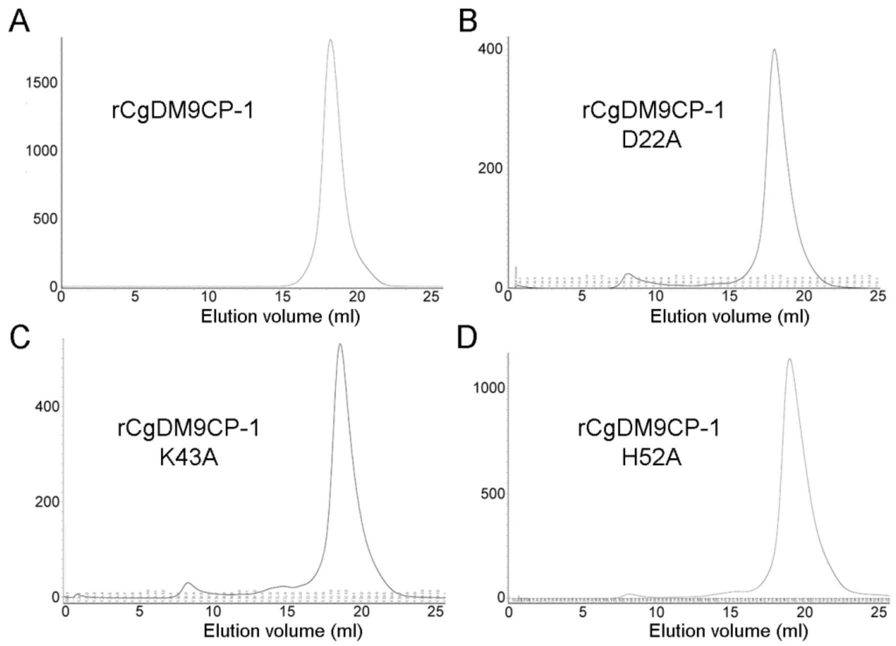

FIGURE S4 Gel filtration chromatography of purified recombinant protein prepared for crystallization. Wild type rCgDM9CP-1 (**A**) and mutants (**B, C** and **D**) were purified by D-mannose-Sepharose 6B affinity chromatography and Ni-NTA affinity chromatography respectively, and further purified by gel filtration chromatography.

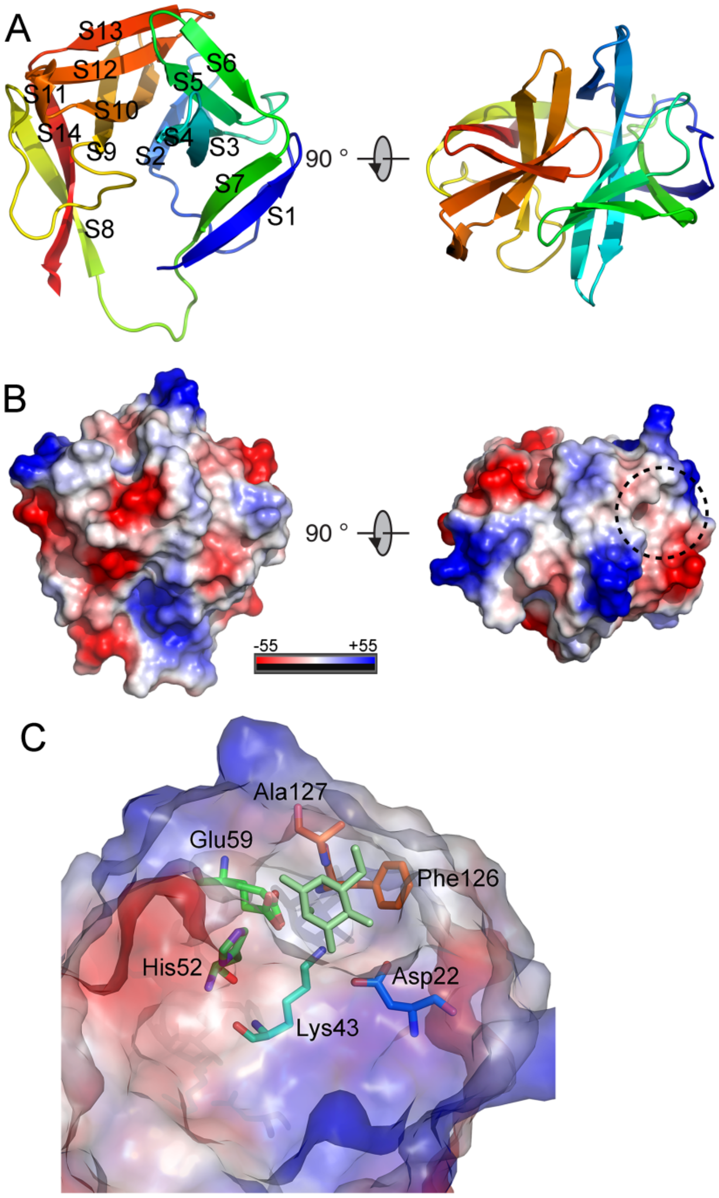

FIGURE S5 The crystal structure of wild type of rCgDM9CP-1 and its complex with D-mannose. (**A**) The overall structure of rCgDM9CP-1 (PDB: 5MH0). Two DM9 domains with β strands from S1 to S7 and from S8 to S14 are related by a pseudo 2-fold axis. (**B**) The electrostatic potential surface onto the rCgDM9CP-1 showing the location of D-mannose binding concave by the dashed lines. (**C**) The D-mannose binding to the rCgDM9CP-1 (PDB: 5MH1) is represented as cyan stick, and the potential amino acid residues involved in the D-mannose binding are shown.

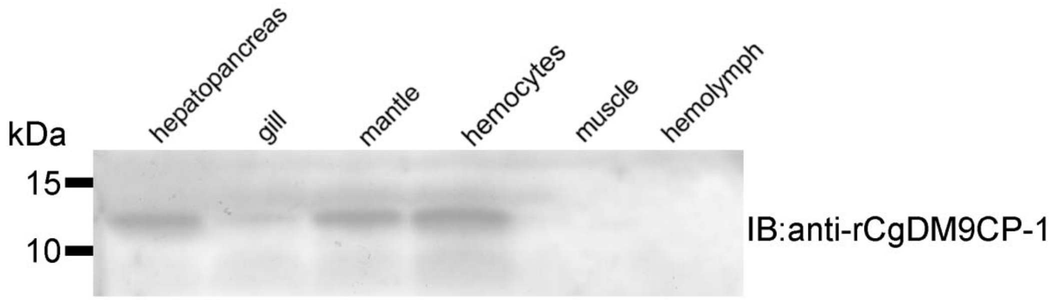

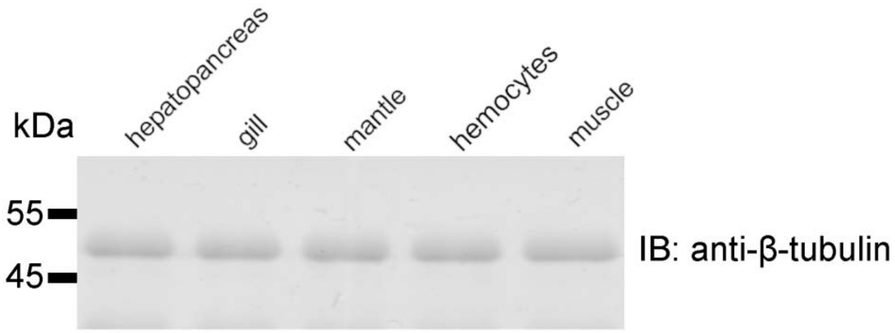

FIGURE S6 The original images of CgDM9CP-1 expression by western blotting presented in Figure 5A.

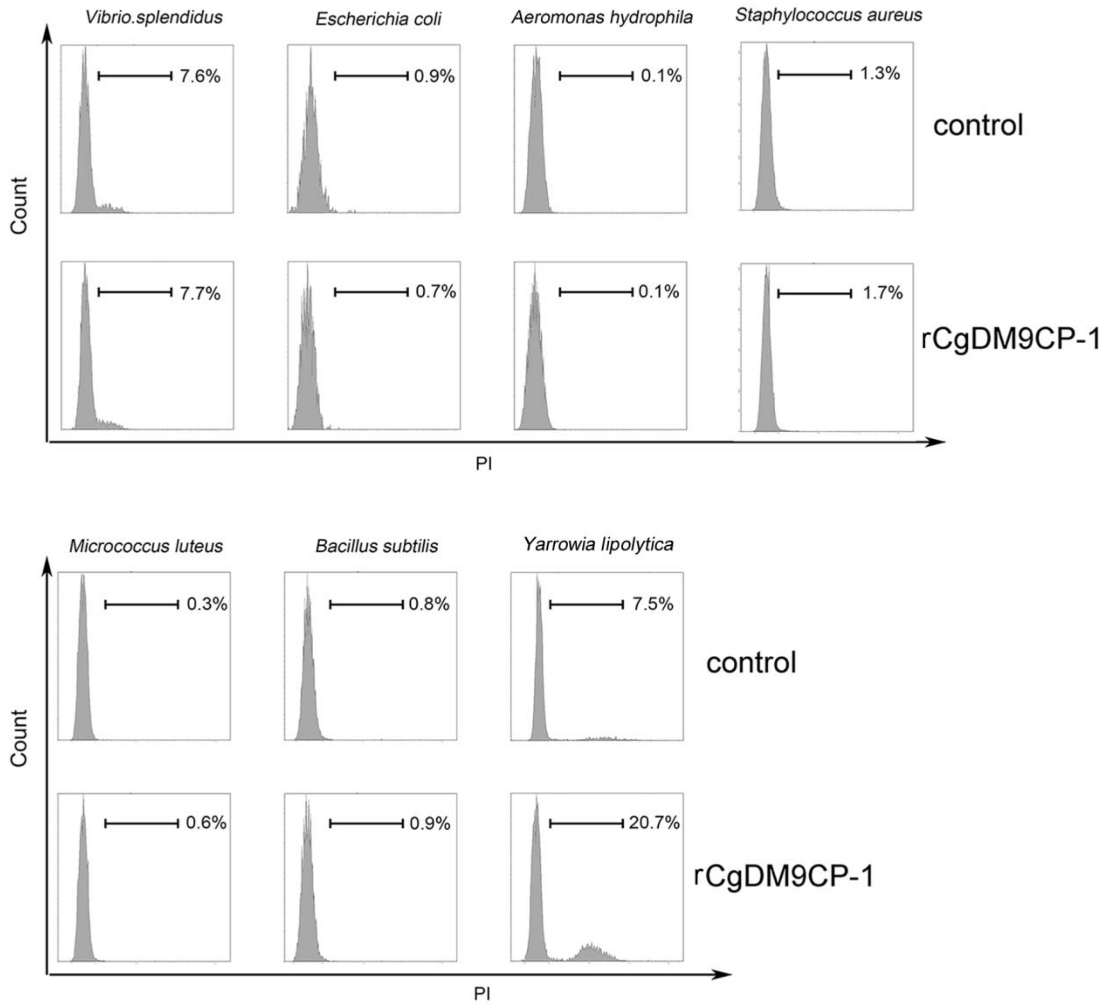

FIGURE S7 rCgDM9CP-1 exhibited antibiotic activity twoards fungi. rCgDM9CP-1 (0.5 mg/ml) were incubated with different microbes for 2 h, followed by propidium iodide staining for 10 min. The antibiotic activity was determined by flow cytometry.

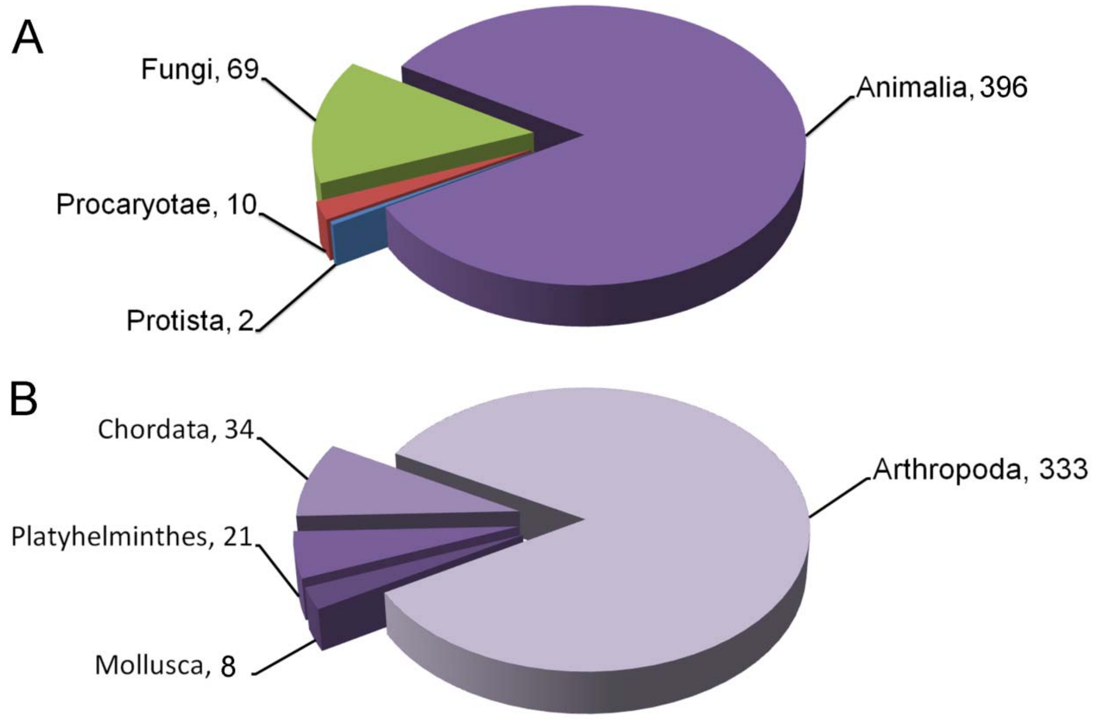

FIGURE S8 The overall distribution of DM9CPs in organisms. (**A**) DM9CPs were identified in Procaryotae, Fungi, Protista and Animalia Kingdom, but not Plantae Kingdom. (**B**) The distribution of DM9CPs in Animalia Kingdom.

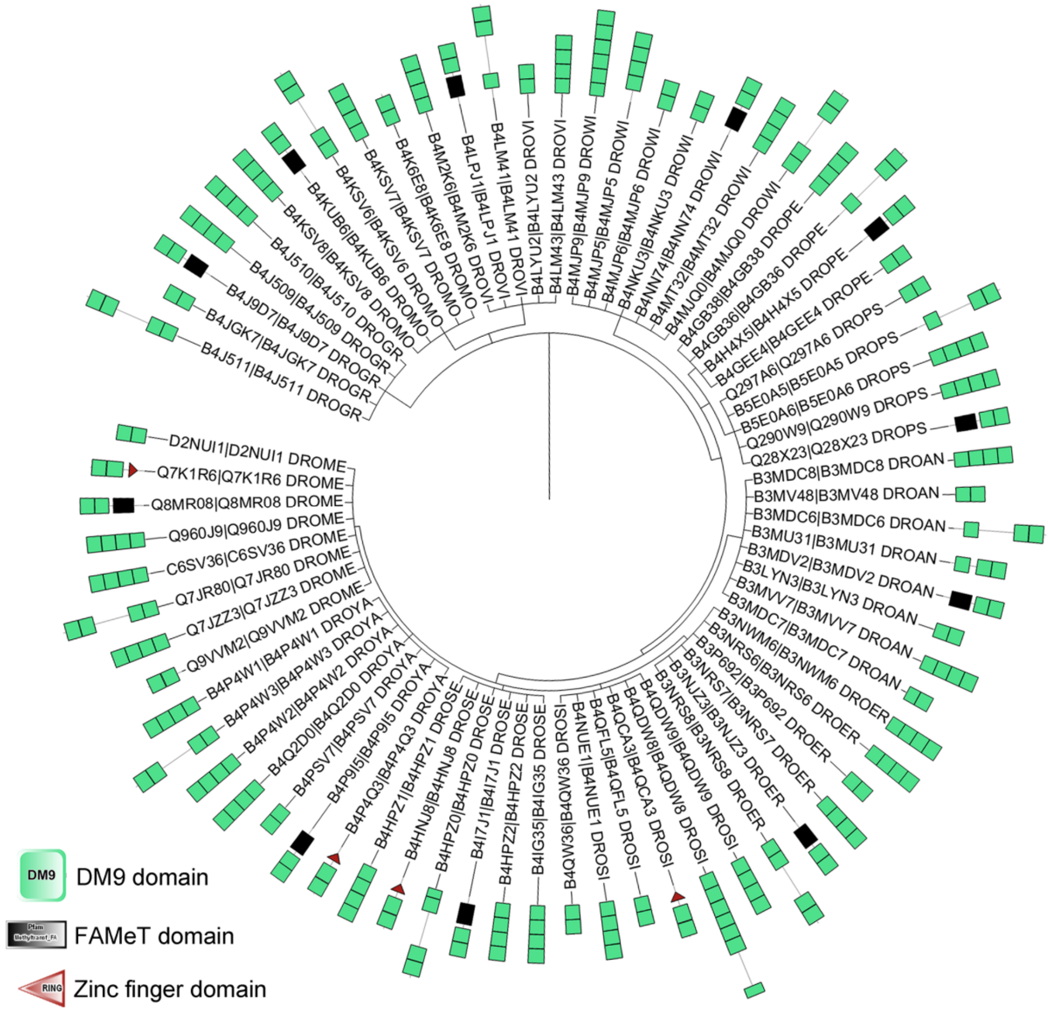

FIGURE S9 Phylogenetic analysis of representative DM9CPs in different *Drosophila* species. DM9 domains are usually found to exist as tandem arranged repeats in proteins from *Drosophila*. The conserved protein domains were identified using the SMART. Circular phylogenetic tree at level of species were created using the iTOL. FAMeT is short for farnesoic acid O-methyl transferase.
