# Supplementary figures and images for: DM9 Domain Containing Protein Functions As a Pattern Recognition Receptor with Broad Microbial Recognition Spectrum
Source: Front Immunol. 2017 Nov 29;8:1607. doi: 10.3389/fimmu.2017.01607 (PMC5712788; doi:10.3389/fimmu.2017.01607)

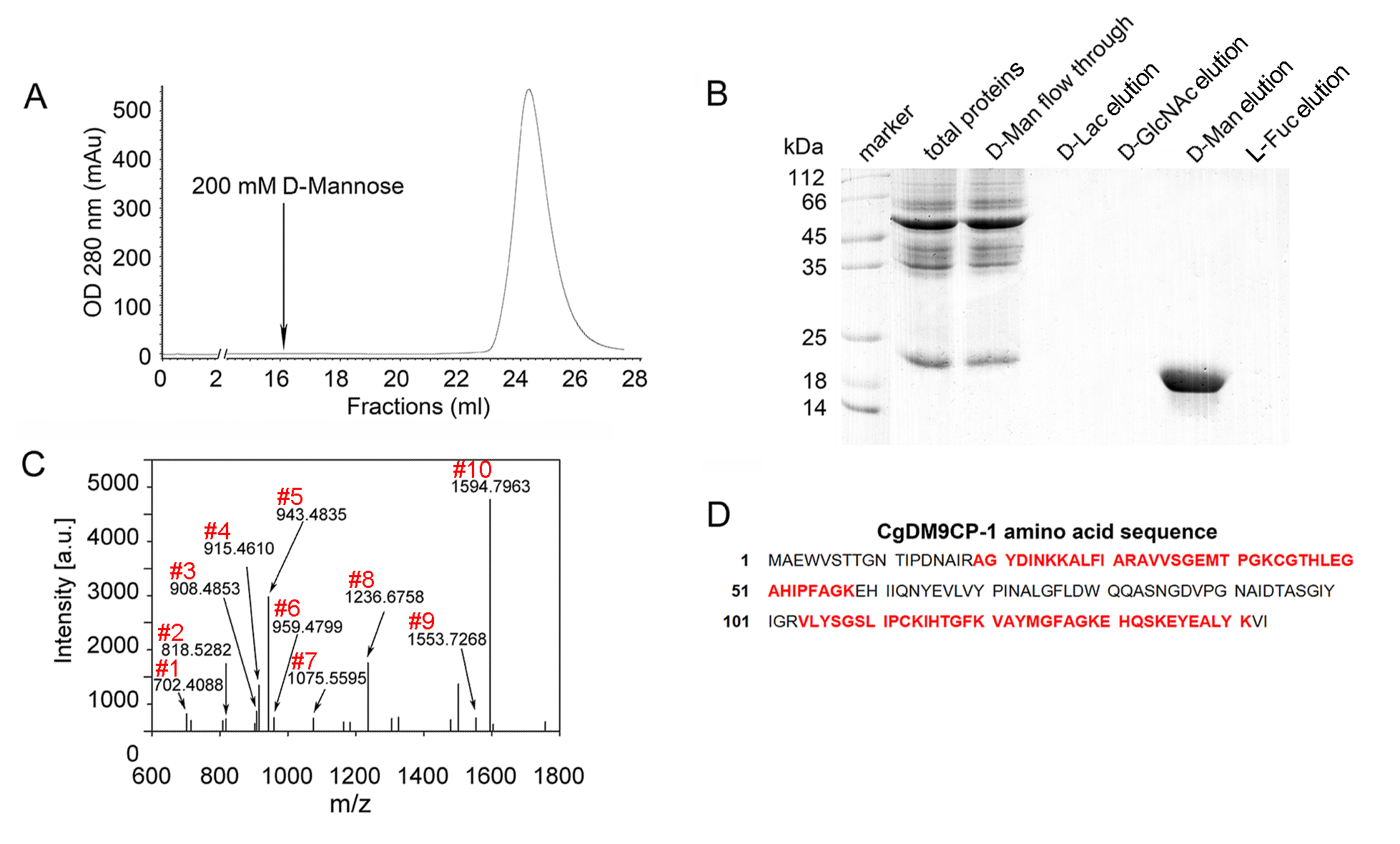

Supplement: Supplementary file 2 [file Image_1.tif]

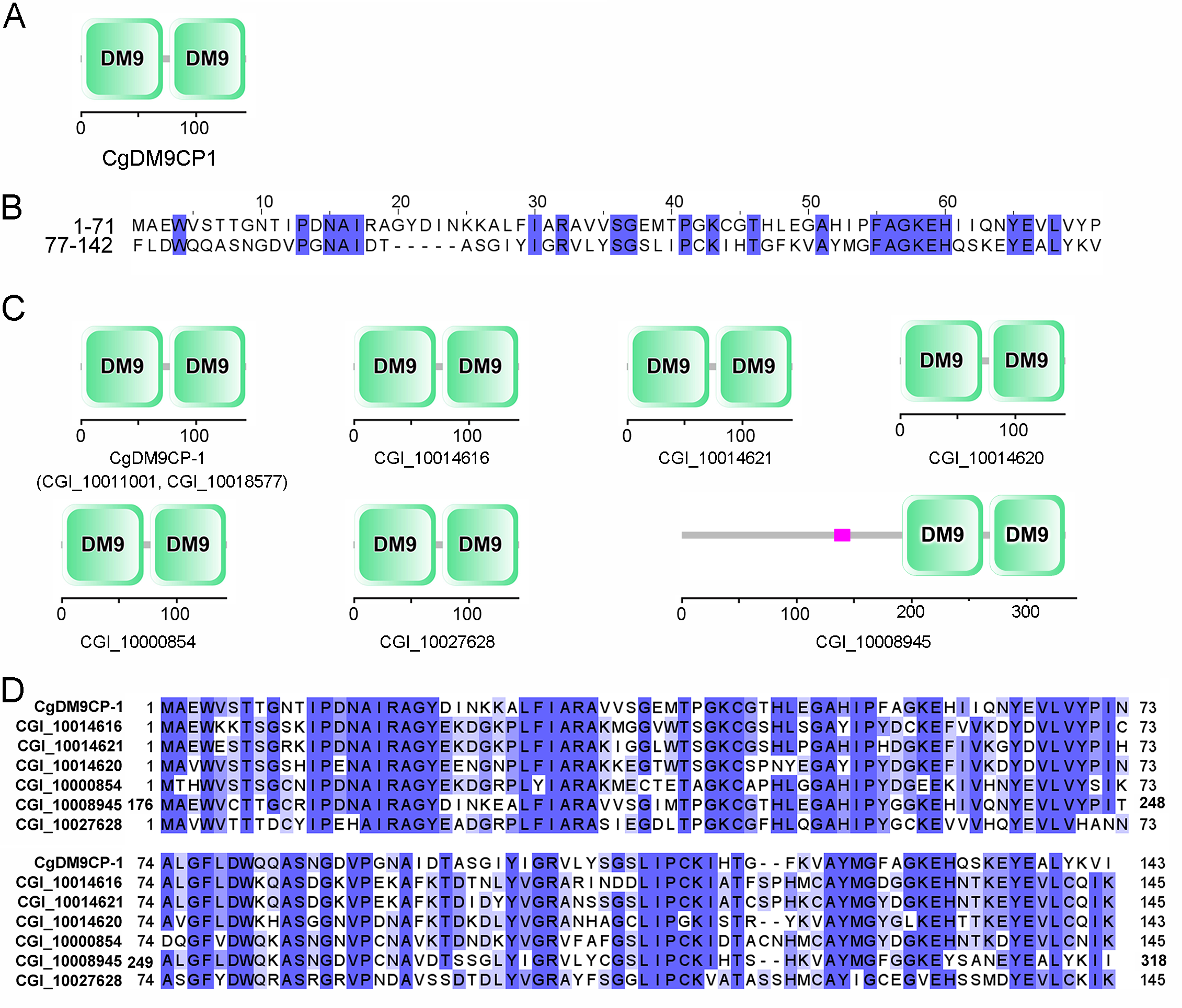

Supplement: Supplementary file 3 [file Image_2.tif]

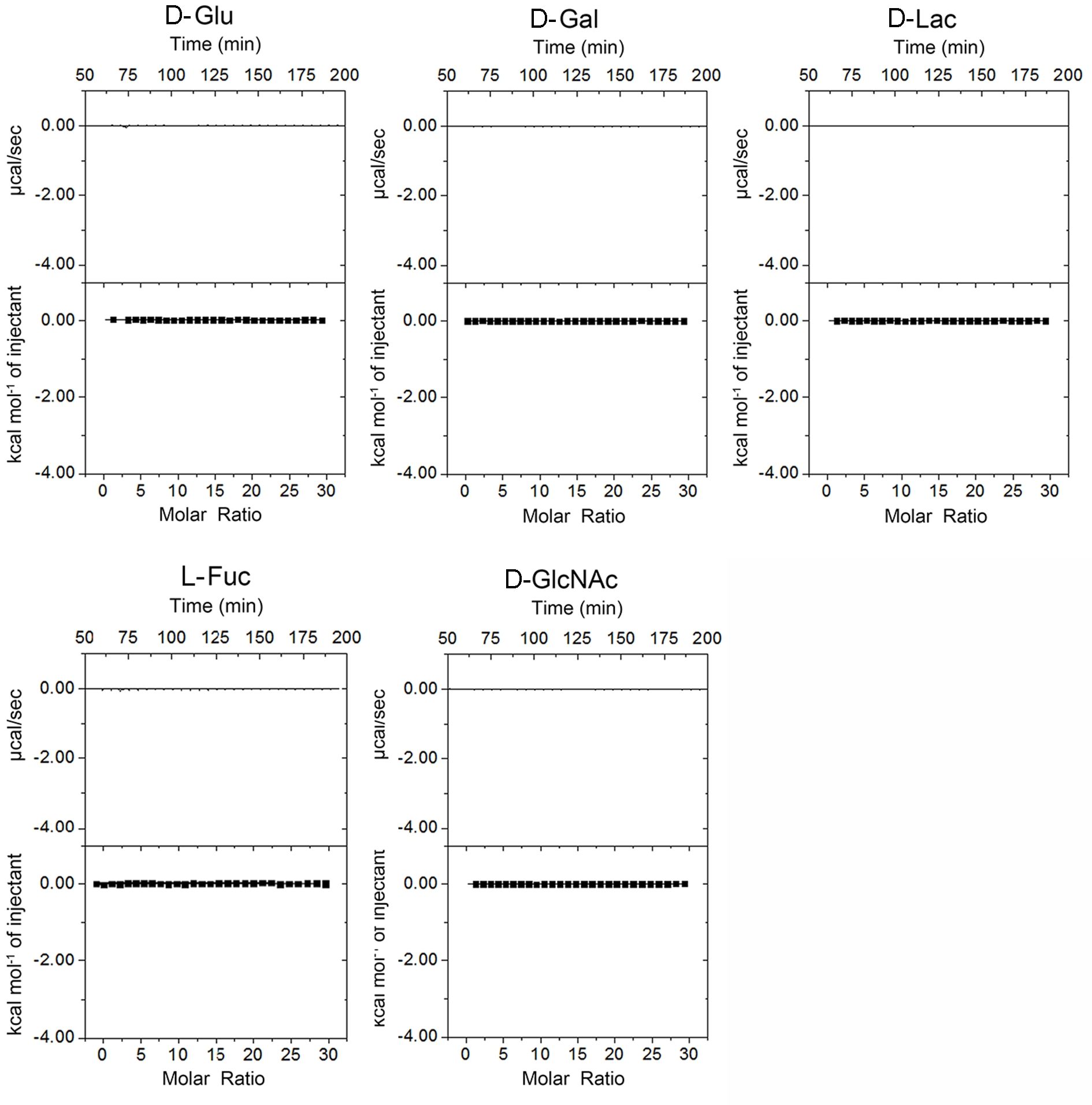

Supplement: Supplementary file 4 [file Image_3.tif]

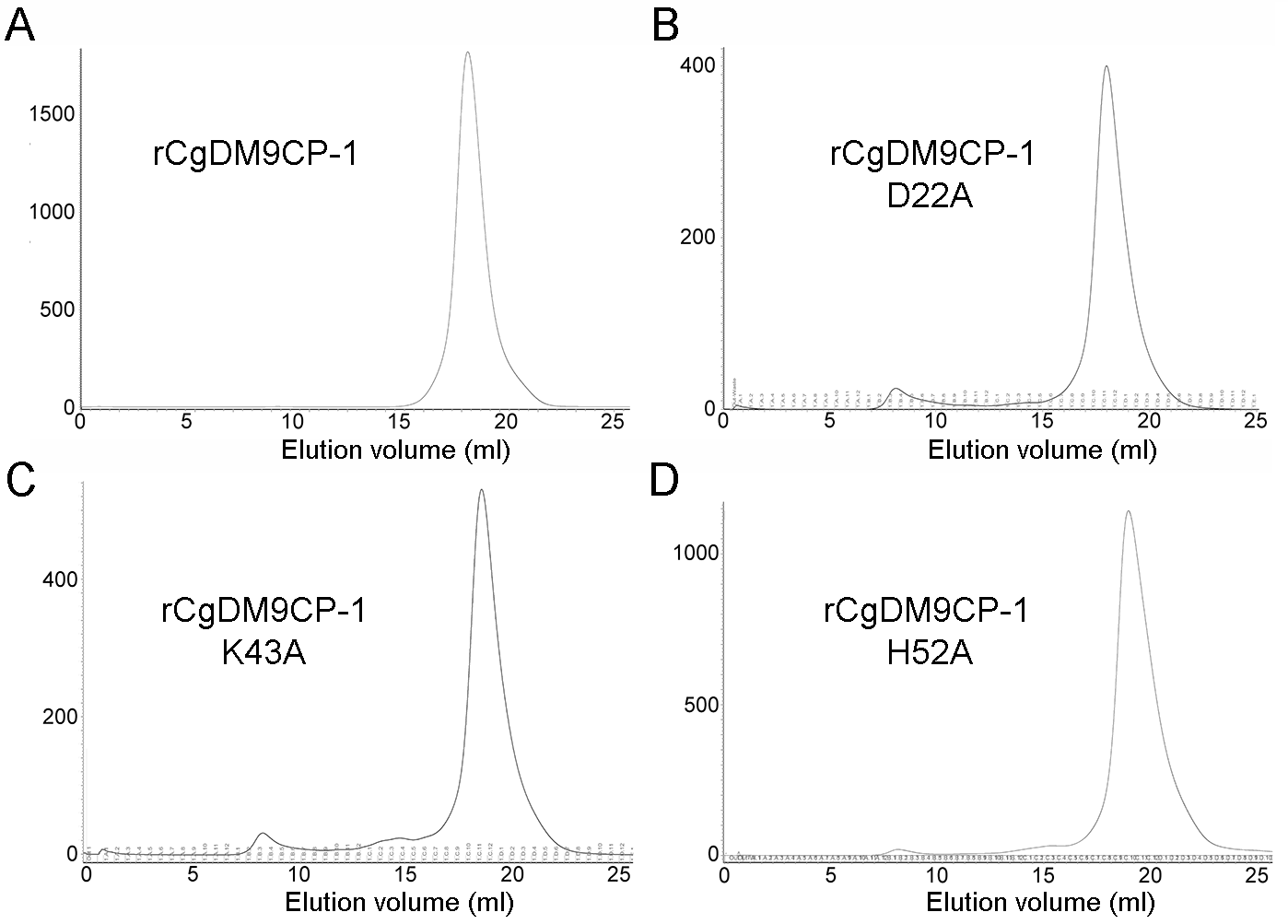

Supplement: Supplementary file 5 [file Image_4.tif]

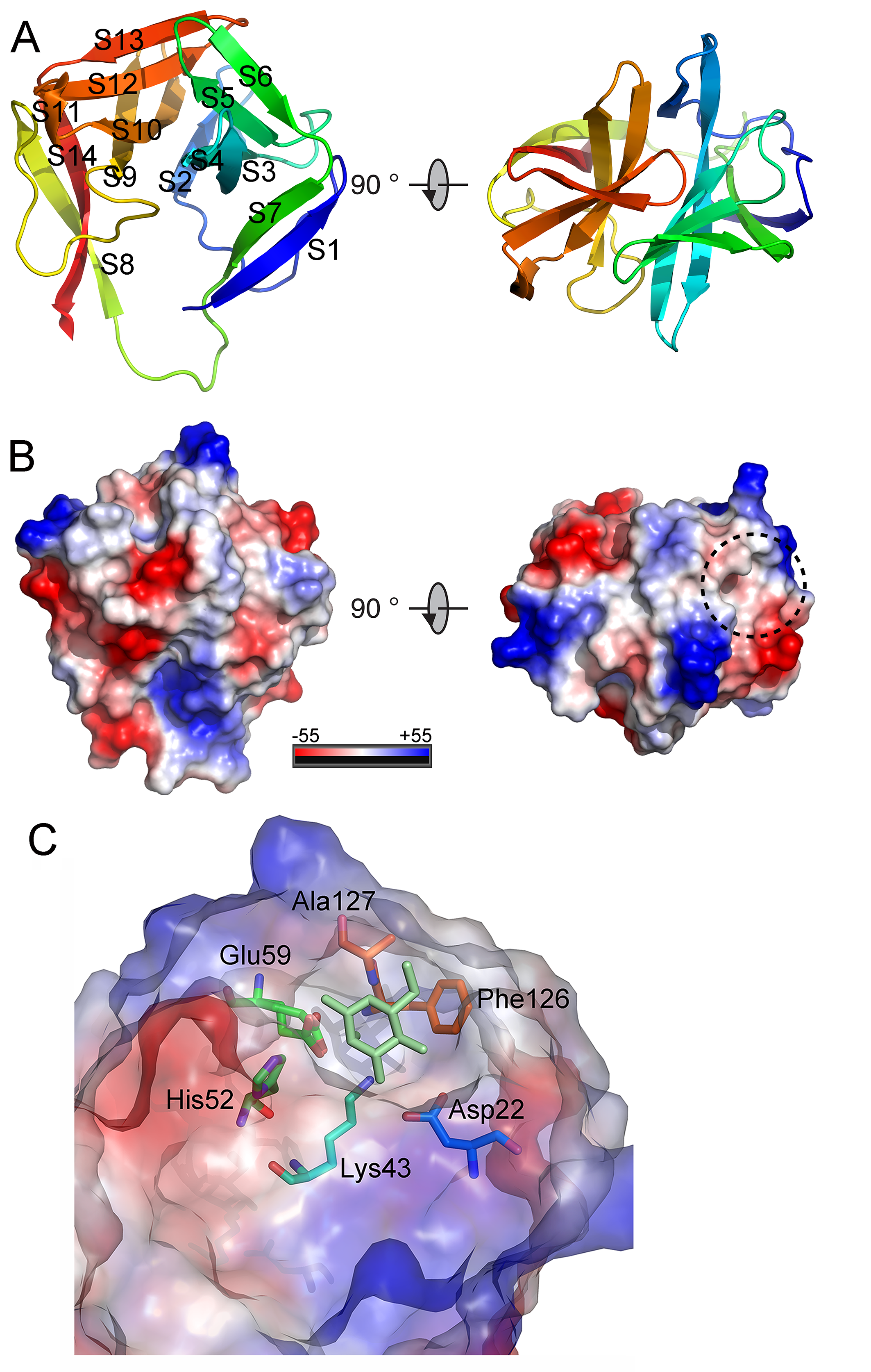

Supplement: Supplementary file 6 [file Image_5.tif]

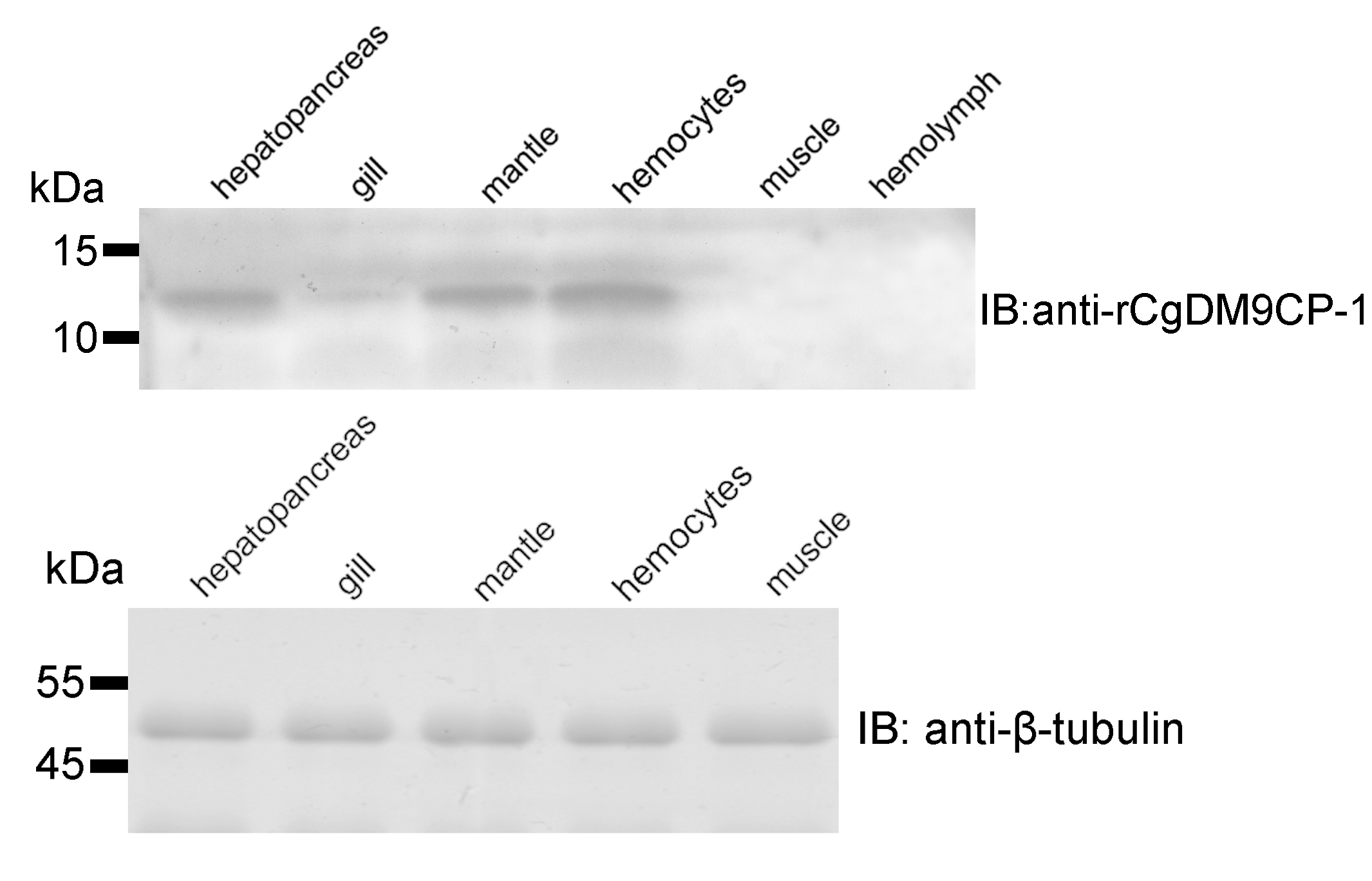

Supplement: Supplementary file 7 [file Image_6.tif]

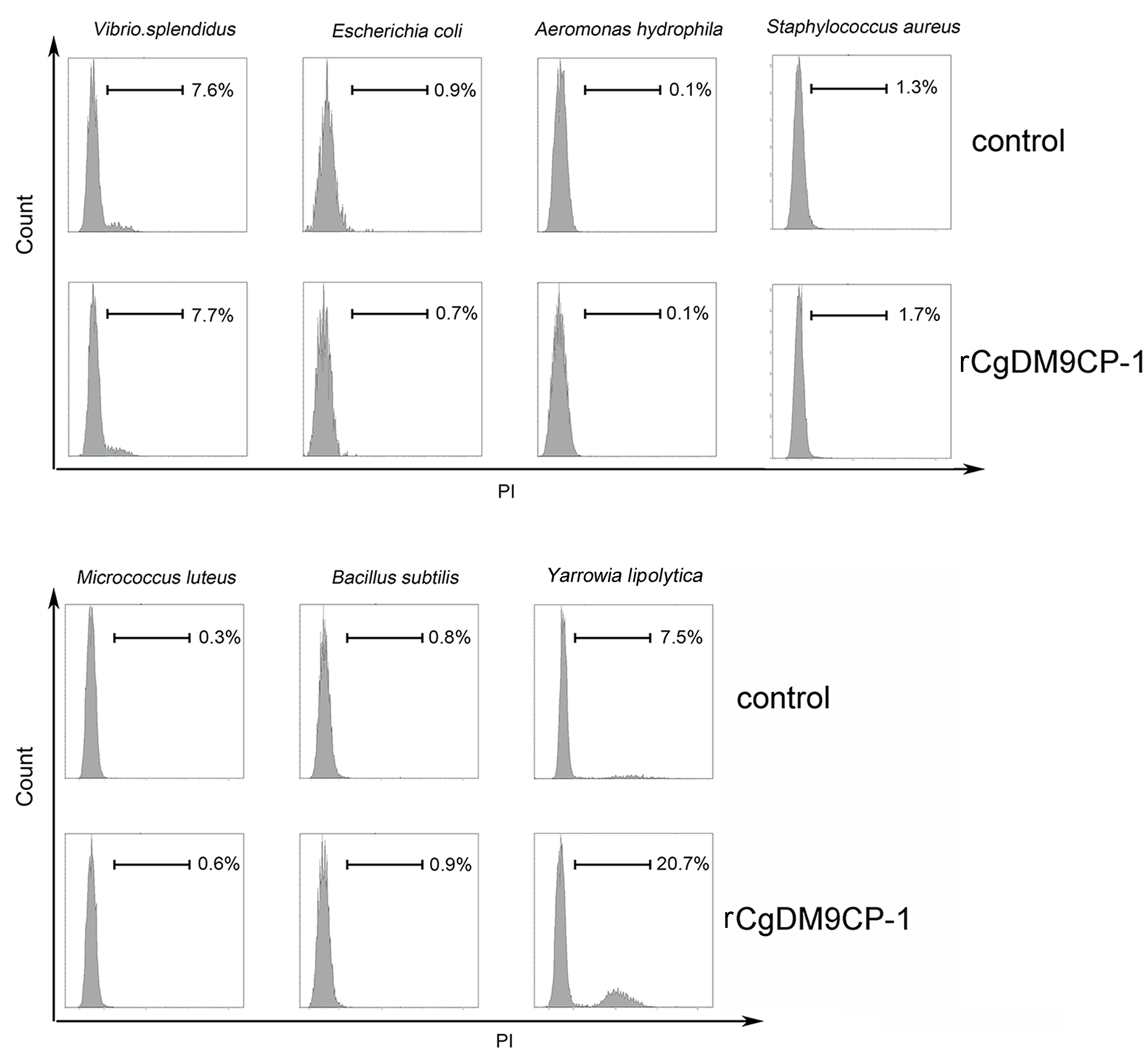

Supplement: Supplementary file 8 [file Image_7.tif]

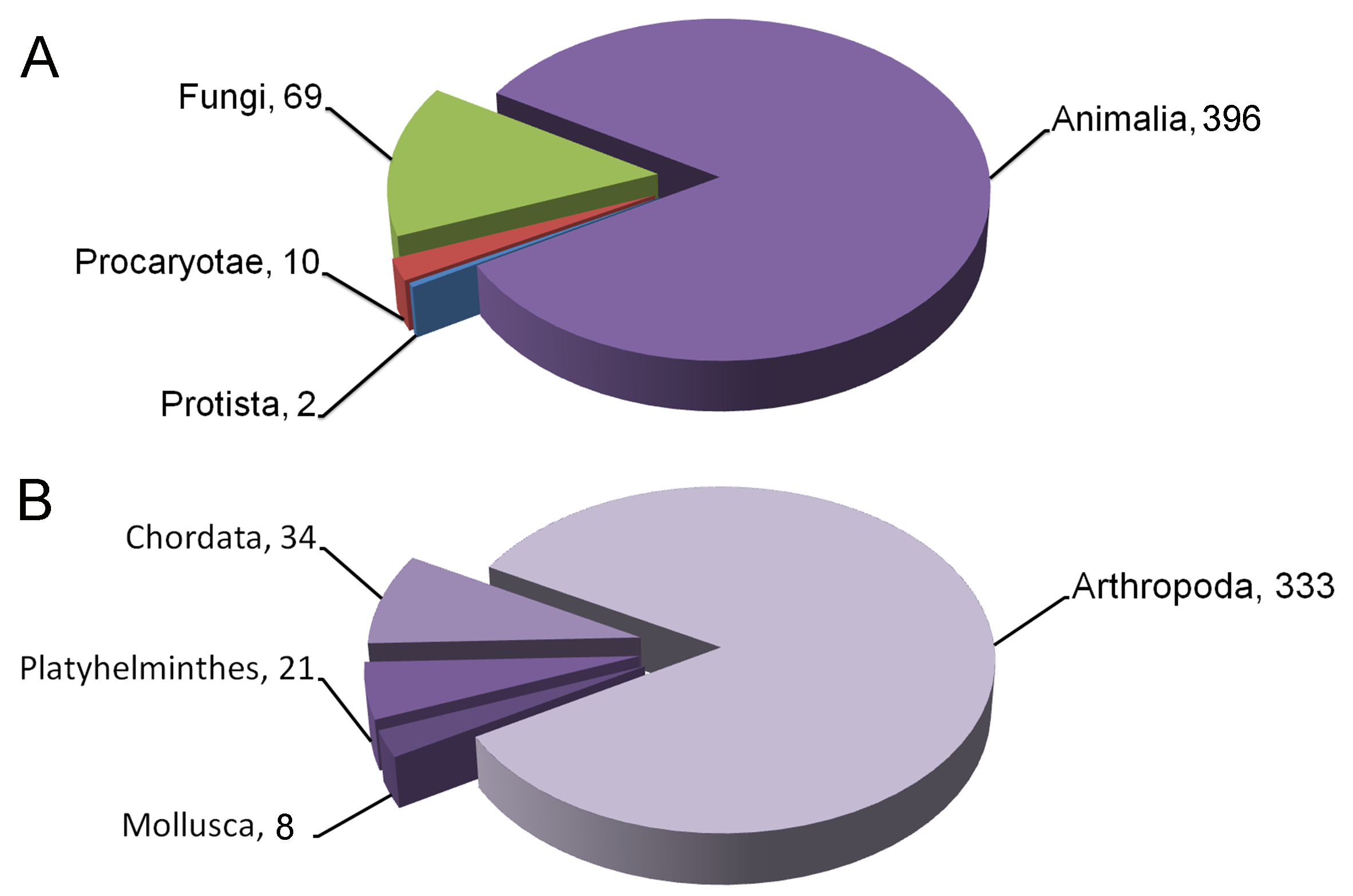

Supplement: Supplementary file 9 [file Image_8.tif]

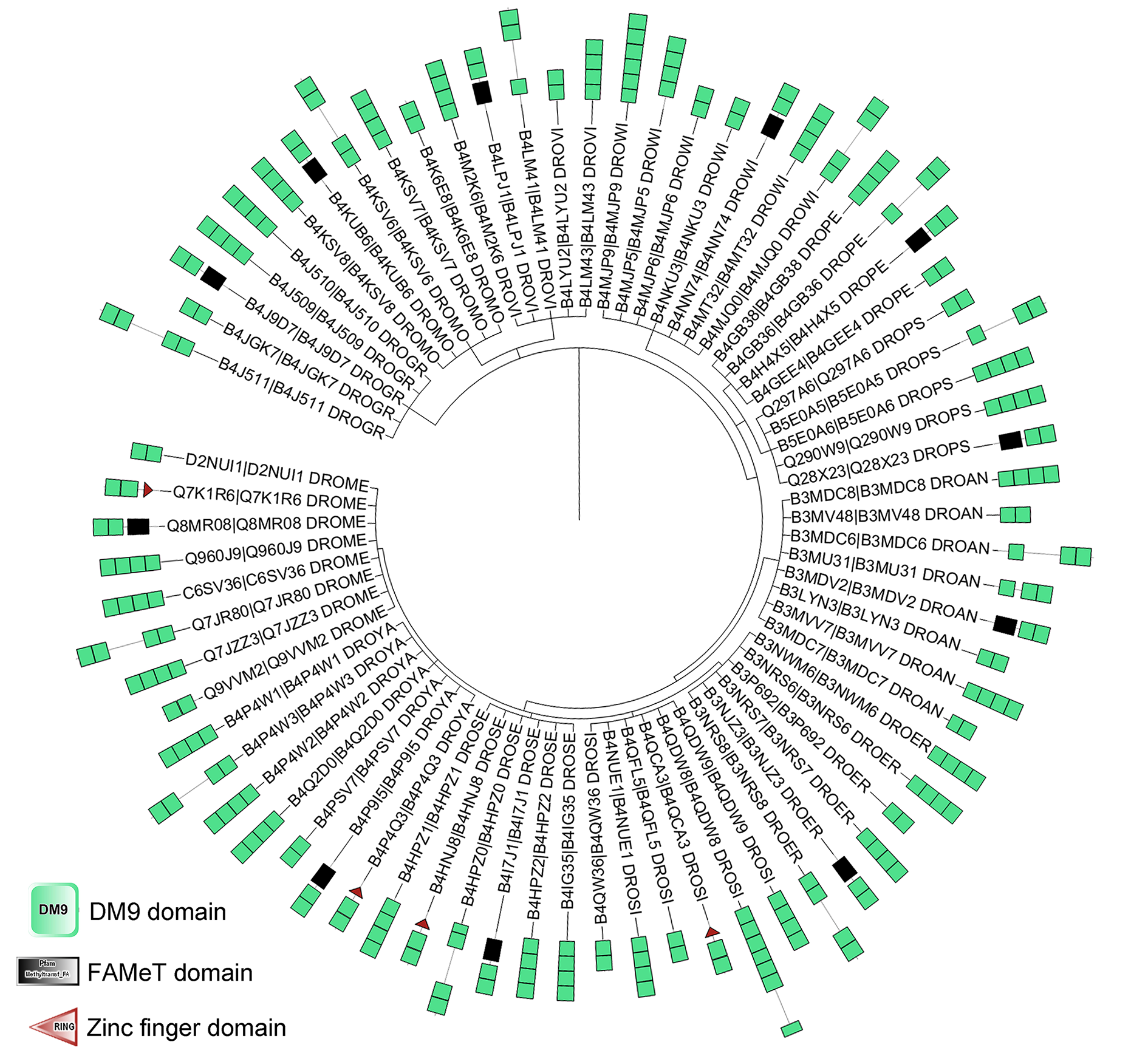

Supplement: Supplementary file 10 [file Image_9.tif]
