# Supplementary material for: DM9 Domain Containing Protein Functions As a Pattern Recognition Receptor with Broad Microbial Recognition Spectrum
Source: Front Immunol. 2017 Nov 29;8:1607. doi: 10.3389/fimmu.2017.01607 (PMC5712788; doi:10.3389/fimmu.2017.01607)
Supplement: Supplementary file 11 [file Table_1.docx]

Table S1. Amino acid sequences of ten tryptic peptides identified by MALDI-TOF/TOF-MS. Peptide #5 and #6 were of the same sequence, whereas methionine of #6 was found to be oxidized.

| Peptides Number | Amino Acid Sequences |
| --- | --- |
| #1 | IHTGFK |
| #2 | KALFIAR |
| #3 | AGYDINKK |
| #4 | EYEALYK |
| #5 | VAYMGFAGK |
| #6 | VAYMGFAGK |
| #7 | AVVSGEMTPGK |
| #8 | VLYSGSLIPCK |
| #9 | VAYMGFAGKEHQSK |
| #10 | CGTHLEGAHIPFAGK |
